# Titanium dioxide nanoparticle impact and translocation through ex vivo, in vivo and in vitro gut epithelia

**DOI:** 10.1186/1743-8977-11-13

**Published:** 2014-03-25

**Authors:** Emilie Brun, Frédérick Barreau, Giulia Veronesi, Barbara Fayard, Stéphanie Sorieul, Corinne Chanéac, Christine Carapito, Thierry Rabilloud, Aloïse Mabondzo, Nathalie Herlin-Boime, Marie Carrière

**Affiliations:** 1 UMR3299 CEA-CNRS, Service Interdisciplinaire des Systèmes Moléculaires et Matériaux, Laboratoire Structure et Dynamique par Résonance Magnétique (LSDRM), CEA Saclay, 91191 Gif sur Yvette, France; 2 Université Paris-Diderot, UMR 843, F75019 Paris, France; 3 INSERM, U843, F75019 Paris, France; 4 European Synchrotron Radiation Facility (ESRF), ID21 beamline, B.P. 220, 38043 Grenoble, France; 5 UMR8502 CNRS-Université Paris Sud, Laboratoire de Physique des solides (LPS), Université Paris-Sud, 91405 Orsay, France; 6 CENBG, Université Bordeaux 1, IN2P3, UMR5797, 33175 Gradignan Cedex, France; 7 UPMC, Univ Paris 06, CNRS, UMR 7574, Chimie de la Matière Condensée de Paris, Collège de France, 11 place Marcelin Berthelot, 75231 Paris Cedex 05, France; 8 Laboratoire de Spectrométrie de Masse BioOrganique (LSMBO), Université de Strasbourg, IPHC, CNRS UMR7178, Strasbourg, France; 9 Pro-MD team, UMR CNRS 5249, Laboratoire de Chimie et Biologie des Métaux, UMR CNRS-CEA-UJF, Grenoble, France; 10 CEA, Direction des Sciences du Vivant, iBiTec-S, Service de Pharmacologie et d’Immunoanalyse, 91191 Gif sur Yvette, France; 11 URA2453 CEA-CNRS, Service des Photons, Atomes et Molécules, Laboratoire Francis Perrin (LFP), CEA Saclay, 91191 Gif sur Yvette, France; 12 Université Grenoble Alpes, INAC, SCIB, F-38000, Grenoble, France 14 CEA, INAC, SCIB, F-38054 Grenoble, France; 13 INSERM U1043, Centre de Physiopathologie de Toulouse Purpan, Université de Toulouse, Toulouse, France

**Keywords:** Titanium dioxide, Nanoparticle, Ingestion, Translocation, Dissolution, Accumulation, Gut, Toxicity, M-cells, Paracellular

## Abstract

**Background:**

TiO_2_ particles are commonly used as dietary supplements and may contain up to 36% of nano-sized particles (TiO_2_-NPs). Still impact and translocation of NPs through the gut epithelium is poorly documented.

**Results:**

We show that, in vivo and ex vivo, agglomerates of TiO_2_-NPs cross both the regular ileum epithelium and the follicle-associated epithelium (FAE) and alter the paracellular permeability of the ileum and colon epithelia. In vitro, they accumulate in M-cells and mucus-secreting cells, much less in enterocytes. They do not cause overt cytotoxicity or apoptosis. They translocate through a model of FAE only, but induce tight junctions remodeling in the regular ileum epithelium, which is a sign of integrity alteration and suggests paracellular passage of NPs. Finally we prove that TiO_2_-NPs do not dissolve when sequestered up to 24 h in gut cells.

**Conclusions:**

Taken together these data prove that TiO_2_-NPs would possibly translocate through both the regular epithelium lining the ileum and through Peyer’s patches, would induce epithelium impairment, and would persist in gut cells where they would possibly induce chronic damage.

## Background

Because of novel properties offered by their small size, nanoparticles (NPs) are introduced in a rising number of commercial products (http://www.nanotechproject.org) including food products [1]. Titanium dioxide (TiO_2_) is the second most used material in consumer products; conventional TiO_2_ is an authorized additive used as a food colorant [1]. Its production was evaluated at 5000 tons/year in 2006-2010 and is estimated to reach 10 000 tons/year in 2011-2014 [2]. In Europe, food-grade TiO_2_ is named E171 [3]; approximately 36% of the particles it contains are less than 100 nm in diameter, i.e. are TiO_2_-NPs [3]. Candies, sweets and chewing-gums have the highest Ti content, i.e. 0.01-1 mg Ti per unit [3]. In sugar-coated chewing gums, the nanoparticulate fraction of TiO_2_ reaches 27.7-43.7% and 95% of TiO_2_-NPs are swallowed upon chewing [4]. Based on consumer intake data, human exposure reaches 1-2 and 0.2-0.7 mg TiO_2_/kg_bw_/day for US children under 10 and other consumers, respectively [3]. Still the literature reporting the impact of TiO_2_-NPs and their translocation through the gut is scarce, as recently reviewed by Bergin et al. [5].

When administered to mice at a single dose of 5 g/kg_bw_, TiO_2_-NPs with diameter 25 or 80 nm translocate through the gut [6]. They distribute through the whole body and particularly in the liver, spleen, kidneys and lungs, showing no overt toxicity but variations in serum biochemical parameters [6]. Upon repeated oral administration at the dose of 12.5 mg/kg during 10 days, 500 nm-TiO_2_ mainly accumulate in gut-associated lymphoid tissues, lungs and peritoneal tissues; they are not detected in the liver and spleen [7]. In young rats, chronic oral administration of 10-200 mg/kg of 75 nm TiO_2_-NPs for 30 days induces liver edema and heart damage as well as cell activation in the stomach. In old rats it causes damage to liver, kidneys and compromises intestinal permeability [8]. Repeated intragastric administration of 5 nm anatase TiO_2_-NPs coated with hydroxypropylmethylcellulose (HPMC) to CD-1 mice causes inflammation and impairs the function of the liver [9,10], kidneys [11] and reproductive system [12]. TiO_2_-NPs are absorbed through the gut and accumulate in internal organs. Conversely, no Ti is detected in the liver, kidneys, spleen and brain of rats exposed by repeated gavage (13-week) of TiO_2_-NPs prepared in water or reconstituted gastric fluid [13]. These contradictory results show that the gut absorption of TiO_2_-NPs depends on the NP preparation procedure and on the in vivo administration protocol.

The gut epithelium is composed of enterocytes, responsible for nutrient absorption, and up to 24% mucus-secreting Goblet cells [14]. The mucus is cytoprotective and represents an efficient physical barrier against pathogens [15]. The most distal part of the ileum presents Peyer’s patches, responsible for gut immunity. This part is also called follicle-associated epithelium (FAE); it is composed of enterocytes and microfold cells (M-cells). M-cells are specialized in the absorption and translocation of large molecules, bacteria and viruses from the intestinal lumen to immune cells.

The uptake of mineral microparticles (i.e. > 100 nm) in the gut mainly occurs through M-cells, while nanoparticles (i.e. <100 nm) are also taken up through enterocytes and goblet cells [7,16-20]. Indeed the apical plasma membrane of mature enterocyte is essentially unable to undergo endocytosis; microvilli morphology per se sterically prevents the invagination of large endocytic vesicles. Consequently mature enterocytes are not able to accumulate and transfer micro- or macro-particles by transcytosis [21]. In rare cases of endocytic events in enterocyte layers, i.e. in the microcrypt areas between neighboring microvilli, endosomes are usually retained in the apical cytoplasm, just beneath microvilli [21]. Goblet cells are still able to undergo endocytosis, and consequently would accumulate nano- and microparticles, but their ability to transfer microparticles by transcytosis has not been reported.

In vitro, most studies reporting the impact of NPs on gut models were performed on Caco-2 cells, either non-differentiated [22,23] or fully differentiated [24,25]. TiO_2_-NPs <40 nm were shown to translocate through differentiated Caco-2 epithelia, causing loss and morphological changes in microvilli, and disorganization of the brush border [25]. Conversely rutile-cored aluminum hydroxide and polydimethylsiloxane-surface-treated TiO_2_-NPs do not accumulate and do not cause any damage in differentiated Caco-2 cells [24]. In undifferentiated Caco-2 cells, TiO_2_-NPs with diameter ranging from 3.94 nm to 25.20 nm induce cell mortality but no DNA damage or oxidative stress [22,23]. Several in vitro studies report particle translocation through a model of FAE consisting in a co-culture of Caco-2 cells and RajiB lymphocytes [26,27], where 15-30% of the Caco-2 cells differentiate into M-cells [26]. Latex beads with diameter 200 and 500 nm are transported through this epithelium [26,27] as well as 200 nm-polystyrene beads [28]. This model was used to characterize the translocation of 30 nm, 70 nm and 112 nm Ag-NPs through the gut. Significant and size-related dissolution of Ag-NPs is reported, leading to the release of Ag^+^ ions. Ag is translocated through this model, as efficiently when Ag-NPs are applied as when Ag^+^ ions are applied [29]. Both Ag-NPs and Ag^+^ ions cause a general stress response in these cells, at the phenotypical and transcriptional levels but this effect is not nano-specific [29]. More recently a tri-culture model, consisting in Caco-2 cells co-cultured with HT29-MTX cells and RajiB cells has been used to assess the impact of polystyrene NPs [30]. Fifty nm NPs are transported through this cell model via the paracellular route while 200 nm NPs are transported via an energy-dependent mechanism i.e. possibly within cellular vesicles. These NPs increase tight junction permeability, due to mechanical disruption of tight junction complexes. Finally, NP exposure increases iron transport through the epithelium by disrupting the cell membrane [30].

In this work we compared the translocation and impact of NPs on various in vivo, ex vivo and in vitro models of regular ileum and FAE. Our aim was to identify the main site and mechanism of NP translocation through the gut. In vitro, we first used a fully differentiated monoculture of Caco-2 cells, which develop a polarized monolayer of enterocytes expressing an organized brush border with a dense network of tight junctions [14]. It is considered a good model for studying the translocation of macromolecules [31]. To reproduce the mucus-secretion of the regular ileum mucosa we used a co-culture of Caco-2 cells with HT29-MTX mucus-secreting cells [32-34]. Finally we used a co-culture of Caco-2 with RajiB cells as an in vitro model of FAE [26,27]. We did not use the tri-culture model described by Mahler et al. [30] because our aim was to compare NP behaviour in the mucus-secreting regular ileum epithelium and in Peyer’s patches that do not produce mucus per se. Ex vivo we used mouse ileum, colon and gut-associated lymphoid tissue mounted in an Ussing chamber [35]; in vivo we exposed mice to a single oral administration of TiO_2_-NPs, at a dose which reflects the daily intake of TiO_2_ by US children. Daily human intake of TiO_2_ from food is in the range of 15-37.5 mg per day for a 75 kg adult. If ~30% of this TiO_2_ is nanoscale then adults would ingest 1.8-4.5 ng of TiO_2_-NPs /cm^2^ of intestine (250 m^2^). We exposed mice to a single gavage of 12.5 mg TiO_2_-NP/kgbw per kg body weight, i.e. 10 ng/cm^2^ of intestine (375 μg TiO_2_-NPs per mice; intestine surface ~2.5 m^2^) which corresponds to 2 to 5-fold the daily intake in an adult human and corresponds to the daily intake in children [3]. This dose can thus be considered realistic. In vitro we chose to expose cells to 50 μg/mL and 2 mL/cm^2^, i.e. 25 μg/cm^2^. This concentration is 10000-fold as high as the daily intake in adult humans, it is thus higher than the dose received by an adult human, but it is classical that such high concentration is applied in vitro; we consider that it reflects a worst case scenario. We used a set of hyphenated microscopy and elemental analysis techniques to assess NP transfer across epithelia and to quantify their accumulation in cells: μ-XRF, μPIXE and TEM observations were carried out, each of them having its own specificity and limit of detection. In addition, in order to identify any dissolution of TiO_2_-NPs in gut cells or during their transfer through gut epithelium, TiO_2_-NP speciation was analyzed by X-ray absorption spectroscopy (XAS) in situ in cells where they were stored. In parallel, we characterized the impact of TiO_2_-NPs on the gut barrier integrity, gut cell cytotoxicity and on the expression of genes encoding proteins involved in epithelial structure.

## Results

### Preparation and characterization of NP suspensions

Our experiments were carried out with a 95% anatase TiO_2_ nanopowder. The mean grain size was 12 ± 3 nm, as measured by TEM. NPs were ultrasonicated in water. The zeta potential of this suspension was 20.0 mV and the average hydrodynamic diameter was 132 nm (Table 1), indicating that some agglomerates and/or aggregates remained in the suspension, as shown in Additional file 1.

**Table 1:** **Physico-chemical characteristics of the TiO**_**2**_**-NP suspensions**^***a***^

**Dispersant**	**Water**	**Gastric**	**Intestinal**	**Ringer + FBS**	**cDMEM**
Z-average (nm)^*b*^	132.0 ± 0.8	218.4 ± 2.9	> 1000	352.0 ± 0.9	320.5 ± 1.8
PdI^*c*^	0.188	0.330	> 0.5	0.298	0.290
ζ (mV)^*d*^	20.0 ± 0.6	30.6 ± 2.7	-21.3 ± 0.7	-13.2 ± 1.2	-10.8 ± 0.6

^*a*^DLS measurements of a 50 μg/mL TiO_2_-NP suspension prepared either in water (“Water”), in the gastric phase (“Gastric” i.e. pepsin/HCl, pH2), in the intestinal phase (“Intestinal”, i.e. pepsin/HCl, pancreatin and bile extracts, pH7), in ex vivo exposure medium (“Ringer + FBS” i.e. Ringer solution, 10% (v/v) fetal bovine serum (FBS), pH7.4) or in in vitro exposure medium (cDMEM, i.e. Dulbecco Modified Medium (DMEM), 10% (v/v) FBS, pH7.4). ^*b*^Z-average hydrodynamic diameter extracted by cumulant analysis of the data. ^*c*^Polydispersity index (PdI) from cumulant fitting. ^*d*^ζ: zeta potential.

We used standard protocols to expose cells, tissues and animals in order to maintain these models in the optimal physiological state. This ensures that we did not induce false positive results that would be due to inappropriate handling of the models. Mice were exposed to a single in vivo gavage of NPs suspended in water. In vivo, these NPs would enter the stomach where the environment is acidic and contains salts and proteins. This environment is classically reproduced in vitro by preparing a gastric juice composed of pepsin, at pH2. When incubated in this in vitro-reconstituted gastric juice, the positive surface charge of TiO_2_-NPs increased, as well as their hydrodynamic diameter and polydispersity index (PdI) (Table 1). This suggests that the stomach juice induced agglomeration and adsorption of proteins on the surface of NPs. We then modelled the entrance of this TiO_2_-NP suspension into the intestine by incubation in a modelled intestinal juice, composed of pancreatin and bile extract at pH7. The zeta potential shifted to a negative value; it reached -21.3 mV. NPs agglomerated as very large clusters (Table 1). Consequently, when entering the gut in vivo, TiO_2_-NPs would be negatively-charged, agglomerated as large clusters and coated with proteins.

Depending on the composition of the meal, fasting/fed mode as well as physiological state upon in vivo exposure, this protein coating would differ. Moreover this coating is dynamic and when transiting through the different gut compartments it would change, as described in a modelled biological system [36]. Since we cannot reproduce all these coatings, we chose to coat NPs with a realistic and biocompatible protein corona. The composition of all body fluids is based on the composition of serum, with addition of some specific proteins. We thus considered that serum was a relevant protein mixture that could model all the protein coronas that would form on the surface of NPs, in vivo.

For the ex vivo experiments, water suspended NPs were diluted in Ringer solution in which we added 10% (vol/vol) of fetal bovine serum (FBS). For in vitro experiments water suspended NPs were diluted in serum-containing cell culture medium (cDMEM for complete DMEM). Upon dilution in these media the zeta potential of TiO_2_-NPs shifted to -13.2 mV and -10.8 mV, respectively (Table 1) and the size of TiO_2_-NP agglomerates increased (Additional file 1). Their Z-average increased to 352 nm and 320.5 nm, and their PdI increased to 0.298 and 0.290 (Table 1), respectively. It suggests that NPs agglomerated. The average diameter of NP agglomerate core, i.e. without the protein layer was 189 ± 87 nm (Additional file 1). The Z-average and PdI were then stable over the next 48 h in these exposure media (Table 2, measurements carried out in the in vitro exposure medium).

**Table 2:** **Stability of the TiO**_**2**_**-NP suspension in serum-containing cell culture medium**^***a***^

**Time after dilution**	**0 h**	**24 h**	**48 h**
Z-average (d, nm)^*b*^	320.5	332.4	323.5
PdI^*c*^	0.290	0.260	0.280
ζ (mV)^*d*^	-10.8 ± 0.6	-11.5 ± 0.3	-10.3 ± 0.5

^*a*^DLS measurements obtained on a 50 μg/mL TiO_2_-NP suspension, immediately after dilution (0 h) or after 24 or 48 h. ^*b*^Z-average hydrodynamic diameter extracted by cumulant analysis of the data. ^*c*^PdI: polydispersity from cumulant fitting. ^*d*^ζ: zeta potential.

Finally we analysed the protein corona that coats the surface of NPs in these in vitro and ex vivo exposure conditions (see Additional file 2). The most abundant proteins in the corona were similar in these two conditions. The protein corona was mainly composed of albumin, apolipoproteins and complement proteins. Still the corona that coated the surface of TiO_2_-NPs in cDMEM was more complex; it contained more than 40 proteins while it contained fewer different proteins in Ringer + FBS.

### Ex vivo and in vivo murine models to assess the transepithelial passage of TiO_2_-NPs

**Figure 1:**
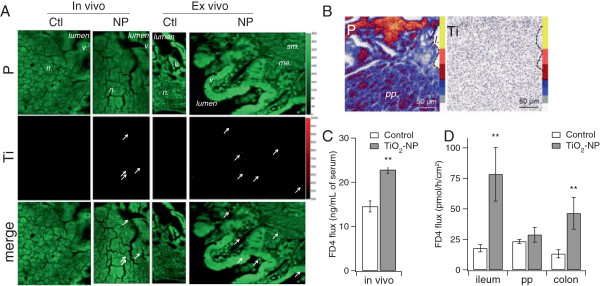
**Micro-XRF observation of TiO**_**2**_**-NP accumulation and impact *ex vivo *and *in vivo*.** In the in vivo experiment, mice were exposed to a single gavage of 12.5 mg/kg TiO_2_-NPs and sacrified 6 h after the gavage (in vivo); ex vivo explants were exposed for 2 h to 50 μg/mL of TiO_2_-NPs. Micro-XRF images of cross sections of the gut from the in vivo (**A**, in vivo) and the ex vivo (**A**, ex vivo) experiments. Green: phosphorous (P) distribution map and red: titanium (Ti) distribution map. Ti-rich zones are pointed out with arrows. PIXE images of a cross section of the gut from the in vivo experiment **(B)**. Phosphorous (P) and titanium (Ti) distribution maps, with their colour scale. Lymphoid nodule (n.), villi (v.), submucosa (sm.), muscularis externa (me.), lumen (l.). Paracellular permability probed by measurement of FITC-Dextran 4 kDa flux through mouse gut in vivo **(C)** and through ex vivo explants of the ileum, Peyer’s patches (pp.) and colon **(D)**. Average of eight replicates ± standard error of the mean. Results were considered statistically significant (**) when p < 0.01.

We monitored Ti transepithelial passage in mice exposed to TiO_2_-NPs by gavage and on mice gut mounted in Ussing chambers. Using μXRF for its high sensitivity and because it makes possible the observation of large areas, we identified Ti-rich regions in regular ileum’s villi as well as in the most peripheral zone of lymphoid nodules (Figure 1A, arrows). These Ti-rich regions were observed both in the in vivo and ex vivo experiment. They were observed in the epithelial layer and deeper in gastro-intestinal tissues, both in the FAE and the regular ileum.

We then imaged the gut sections of in vivo NP-exposed mice by μPIXE (Figure 1B). The advantage of μPIXE is that it gives quantitative information, contrary to μ-XRF. However the limit of detection of μPIXE is 20-30 ppm while it is 1-2 ppm in μXRF. Unfortunately, the Ti content in the in vivo and ex vivo samples was too low to be detected and quantified by μPIXE.

Finally since the translocation of some NPs, e.g. Ag-NPs, have been partly attributed to their dissolution and passage of metal ions rather than NPs [29], we assessed TiO_2_-NP dissolution in gut tissues. In this purpose we carried out in situ X-ray absorption spectroscopy analyses (XAS) of the Ti-rich regions detected in μXRF images. Unfortunately Ti content in these Ti-rich regions was too small to be adequately analysed by XAS.

The literature shows that microparticles -and thus potentially the TiO_2_-NP agglomerates that we used in this study- would only pass through the gut epithelium via M-cells. An explanation of their passage through the regular ileum epithelium would be that tight junctions are disrupted. To test this hypothesis we measured gut paracellular permeability in the in vivo and ex vivo models exposed to TiO_2_-NPs. As probed by FITC-Dextran 4 kDa flux, TiO_2_-NP exposure increased the paracellular permeability of mice gut, in the vivo model (Figure 1C). It also increased the paracellular permeability, ex vivo, in the ileum and colon but not in Peyer’s patches (Figure 1D). Finaly we measured the relative expression of genes encoding proteins involved in the maintenance of cell junctions. The expression of genes encoding zonula occludens protein 1 and 2 (TJP1 and TJP2, also termed ZO-1 and ZO-2), claudin 2 and 3 (CLDN2 and CLDN3) and occludin (OCLN) were down-regulated in the ileum of TiO_2_-NP-exposed mice, but not in the colon, while the expression of claudin 5 (CLDN5) remained unchanged (Table 3). The integrity of the ileum epithelium was thus compromised by TiO_2_-NP exposure. It suggests that TiO_2_-NPs would possibly pass through the regular ileum by following the paracellular route via disrupted tight junctions.

**Table 3:** **In vivo expression of genes encoding junction proteins**^***a***^

	**CLDN2**	**CLDN3**	**CLDN5**	**OCLN**	**TJP1**	**TJP2**
Ileum Ctl	1.02 ± 0.17	1.07 ± 0.39	1.05 ± 0.33	1.03 ± 0.23	1.08 ± 0.39	1.06 ± 0.33
Ileum NP	0.74 ± 0.37*	1.26 ± 0.60*	1.64 ± 1.07	0.72 ± 0.32*	1.54 ± 0.58*	1.27 ± 0.53*
Colon Ctl	n/a	1.02 ± 0.19	n/a	n/a	1.11 ± 0.06	1.01 ± 0.16
Colon NP	n/a	0.61 ± 0.16	n/a	n/a	0.75 ± 0.16	0.69 ± 0.18

^*a*^Relative expression of genes encoding proteins involved in cell junction maintenance, measured in the ileum and colon of control mice (not exposed to NPs) and mice exposed to a single gavage of 12.5 mg of TiO_2_-NP/kgbw and sacrificed after 6 h, n/a not measured. Results are the average of 6 replicates ± standard deviation. Statistical significance was examined by randomization tests using REST2009 [37].

### Human In vitro models of gut

#### Cell viability and impact of NPs

**Figure 2:**
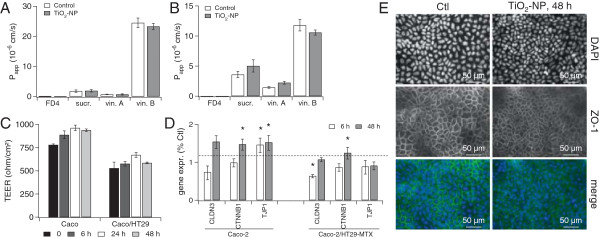
**Impact of TiO**_**2**_**-NPs on *in vitro *gut epithelia transport function and integrity.** Transport functions of Caco-2 cells **(A)** or Caco-2/HT29-MTX coculture **(B)** exposed to 50 μg/mL TiO_2_-NPs for 48 h (TiO_2_-NP) or not exposed (Control). Paracellular transport is probed by FITC-Dextran 4 kDa (FD4) and ^14^C-sucrose (sucr.) fluxes; the activity of the P-gP is probed by ^14^C-vinblastin apical to basolateral (vin. A) or basolateral to apical (vin. B) fluxes. Papp is the permeability coefficient, calculated as P_app_ = dQ/(dt × A × C_0_) where dQ/dt is the amount of labelled compound transported per time unit; A is the surface of the transwell and C_0_ is the initial concentration in the donor compartment. Transepithelial resistance (TEER) of Caco-2 cells and Caco-2/HT29-MTX coculture exposed for 0 h, 6 h, 24 h or 48 h to 50 μg/mL of TiO_2_-NPs **(C)**. Relative expression of genes encoding proteins involved in cell junction maintenance, measured in cells exposed to 50 μg/mL of TiO_2_-NPs for 6 h or 48 h **(D)**. Immunstaining of the nucleus (DAPI) and ZO-1 (TJP1) protein distribution in Caco-2 cells either non exposed (Ctl) or exposed to 50 μg/mL TiO2-NPs for 48 h **(E)**. Results are the average of three replicates ± standard deviation; they were considered statistically significant (*) when p < 0.05 **(A-C)**. Statistical significance of qPCR data **(D)** was examined by randomization tests using REST2009 [37].

We assessed the impact of NPs on three in vitro models of gut epithelium: a monoculture of Caco-2 cells (regular epithelium), a co-culture of Caco-2 and HT29-MTX cells (mucus-secreting regular epithelium) and a coculture of Caco-2 and RajiB cells (FAE). The characterization of these cell models is described in the Additional file 3. Exposure to 50 μg/mL TiO_2_-NPs for 48 h did not alter the paracellular permeability and transport through P-glycoprotein in Caco-2 (Figure 2A) and Caco-2/HT29-MTX (Figure 2B) models, assessed by quantification of FITC-Dextran and radiolabelled sucrose fluxes (paracellular permeability) and vinblastin flux (transport through p-glycoprotein). Neither did it induce any significant loss of TEER in both models, proving that TiO_2_-NPs did not disrupt the junctions of these epithelia (Figure 2C). However we measured significant, although moderate, modulations in the expression of genes encoding proteins involved in the maintenance of cell junctions, both in the Caco-2 and Caco-2/HT29-MTX models exposed to TiO_2_-NPs (Figure 2D). We observed upregulation of TJP1 (ZO-1, a component of tight junctions) in Caco-2 cells after 6 h and 48 h of exposure and upregulation of CTNNB1, encoding β-catenin (cadherin-associated protein, part of a complex of proteins constituting adherens junctions) in both cell models after 48 h of exposure. CLDN3 (component of tight junction strands) was downregulated after 6 h of exposure of the Caco-2/HT29-MTX coculture. Finally upregulation of TJP1 did not lead to the redistribution of TJP1 protein in tight junctions (Figure 2E).

**Figure 3:**
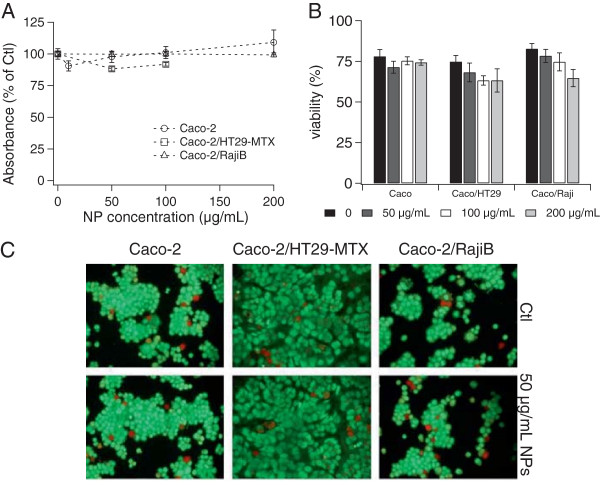
**NP impact on cell viability and epithelial integrity.** Cell viability probed with the MTT assay **(A)** or trypan blue exclusion assay **(B)** after 48 h of exposure to TiO_2_-NP concentrations ranging from 0 to 200 μg/mL. Results are expressed as average of eight replicates (MTT) or three replicates (trypan blue) ± standard deviation; they were considered statistically significant (*) when p < 0.05. Caco: Caco-2, HT29: HT29-MTX. Assessment of apoptosis and necrosis in control epithelia or epithelia exposed for 48 h to 50 μg/mL of TiO_2_-NPs **(C)**: cells were stained with acridin orange/ethidium bromide; viable cells stain green, apoptotic cells stain orange and necrotic cells stain red.

Since NPs may translocate through free routes left by dead cells in the epithelial layer, we assessed cell mortality and apoptosis caused by TiO_2_-NPs. Exposure to 10-200 μg/mL of NPs did not cause overt cytotoxicity, as probed using the MTT and trypan blue exclusion assays (Figure 3A-B). The interference of NPs with the MTT assay was characterized (see Additional file 4). No apoptosis was observed, as shown by acridin orange/ethidium bromide staining (Figure 3C, live cells appear green, apoptotic cells appear orange/yellow and necrotic cells appear red).

#### Quantification of NP uptake

**Figure 4:**
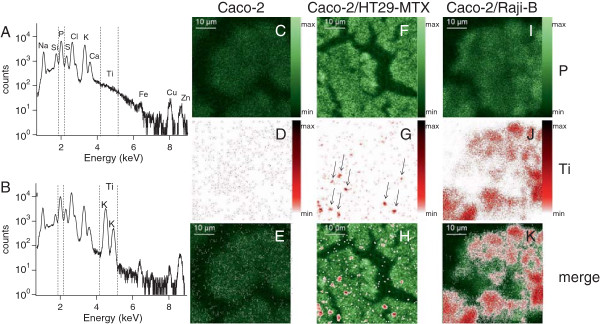
**Particle-induced X-ray emission (PIXE) spectra and maps of P and Ti distribution.** PIXE spectra displaying the regions of interest (between dashed lines) selected for mapping and quantification of P and Ti content, on Caco-2 monoculture **(A)** and Caco-2/HT29-MTX co-culture **(B)**. Chemical element distribution in the Caco-2 monoculture **(C-E)**, Caco-2/HT29-MTX co-culture **(F-H)** and the Caco-2/RajiB co-culture **(I-K)**, exposed to 50 μg/mL TiO_2_-NPs for 24 h: distribution of phosphorous (P) **(C, F, I)** and of titanium (Ti) **(D, G, J)**, merge of P and Ti distribution maps **(E, H, K)**.

We then evaluated TiO_2_-NP accumulation in the three in vitro models, by using μPIXE. TiO_2_-NP accumulation in Caco-2 monocultures was very low: PIXE spectra recorded on this model showed no clear Ti line (Figure 4A). Conversely the PIXE spectra recorded on the Caco-2/HT29-MTX co-culture showed two intense Ti lines, corresponding to the Ti-Kα and -Kβ emissions (Figure 4B). Images of Ti distribution logically showed very few Ti-rich areas in the Caco-2 monoculture (Figure 4C-E), while Ti-rich areas were observed in the Caco-2/HT29-MTX co-culture (Figure 4F-H, arrows). Ti accumulation was also significant in the Caco-2/RajiB coculture (Figure 4I-K). It suggests that the presence of HT29-MTX cells throughout Caco-2 monolayer, as well as partial differentiation of Caco-2 cells into M-cells allowed TiO_2_-NPs to be accumulated in cells. Ti distribution images were also recorded on cell cross-sections (Additional file 5), proving that the Ti-rich areas were really inside the cells, and not deposited on their surface.

Ti content was quantified by integrating PIXE spectra of the Ti-rich intracellular areas. It was normalized with respect to phosphorous (P) content (Table 4). The highest TiO_2_-NP accumulation was observed in the Caco-2/RajiB model. In this model, it was 1.8-fold as high as in the Caco-2/HT29-MTX model. In the Caco-2 monoculture, TiO_2_-NP accumulation was insignificant. TiO_2_-NPs thus accumulated both in the regular ileum epithelium model and in the FAE model, in vitro.

**Table 4:** **Ti/P content in the three epithelial models**^***a***^

**Cell model**	**Ti/P**^***b***^
Caco-2 monoculture	0.020 ± 0.012
Caco-2/HT29-MTX coculture	0.108 ± 0.047
Caco-2/RajiB coculture	0.183 ± 0.039

^*a*^Ti and P content were measured locally, by μPIXE, in Ti-rich regions detected in Ti distribution maps, in cells exposed to 50 μg/mL of TiO_2_-NPs for 24 h. ^*b*^Ratio of titanium (Ti) and phosphorous (P) concentrations.

**Figure 5:**
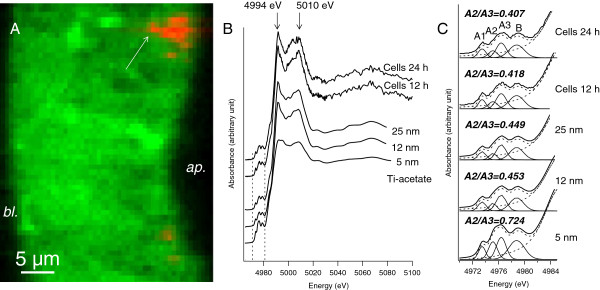
**In situ XAS analysis of intracellular Ti-rich regions.** Micro-XRF image of a cross-section of Caco-2 cells exposed for 24 h, on their apical pole, to 50 μg/mL of TiO_2_-NPs **(A)**. Ap.: apical pole; bl.: basolateral pole. Phosphorous (P) distribution map is depicted in green and titanium (Ti) distribution map is depicted in red. The area pointed out with an arrow was further analysed by XAS. XAS spectra of reference Ti-acetate and TiO_2_ anatase nanopowders (5, 12 and 25 nm) and of Ti-rich regions in Caco-2 cells exposed for 12 h (Cells 12 h) or 24 h (Cells 24 h) to 50 μg/mL of 12 nm-diameter anatase TiO_2_-NPs **(B)**. Focus on the pre-edge region (4972-4985 eV) and its deconvolution using an arctangent function and 4 Gaussian peaks (A1, A2, A3, B) **(C)**. Solid line: recorded data, dashed line: fit. Panels indicate A2/A3, which is the ration of intensity of A2 to intensity of A3.

We then analysed Ti speciation in cells in order to detect any dissolution of TiO_2_-NPs. In this purpose we imaged Ti distribution by μXRF in cross sections of NP-exposed cells (Figure 5A) and analysed the Ti-rich areas by XAS, in situ. The XAS spectra obtained in cells exposed for 12 h or 24 h to TiO_2_-NPs were very similar to the spectrum obtained on the 12 nm diameter TiO_2_-NPs nanopowder used to expose the cells (Figure 5B), with two characteristic sharp lines at 4994 eV and 5010 eV. This proves that the Ti detected in cells was from anatase TiO_2_-NPs (for comparison with rutile TiO_2_-NPs, see [38]) and not from dissolved Ti ions. Chemical composition (mainly Ti and O) and crystalline structure were confirmed by electron dispersive spectroscopy and electron diffraction analysis, carried out on TEM grids prepared from the same samples (see Additional file 6). Moreover, the shape of the pre-edge region of XAS spectra (4972-4985 eV) varies with the diameter of TiO_2_-NPs [39-41]. The shape of this region was not significantly modified, as measured by the relative intensity of A2 to A3 lines (Figure 5C). Consequently TiO_2_-NP storage for up to 24 h in cells did not induce any decrease of NP diameter, i.e. did not induce any dissolution of TiO_2_-NPs. To confirm this result, we measured Ti content in centrifuged lysates of TiO_2_-NP-exposed cells by ICP-MS. The centrifugation procedure enables cell debris and nanoparticles to be accumulated in the pellet, while the cytosol which possibly contains dissolved Ti ions remains in the supernatant. This procedure also failed to detect any dissolved Ti ions in the cell cytosol (not shown).

#### Evaluation of NP translocation

**Figure 6:**
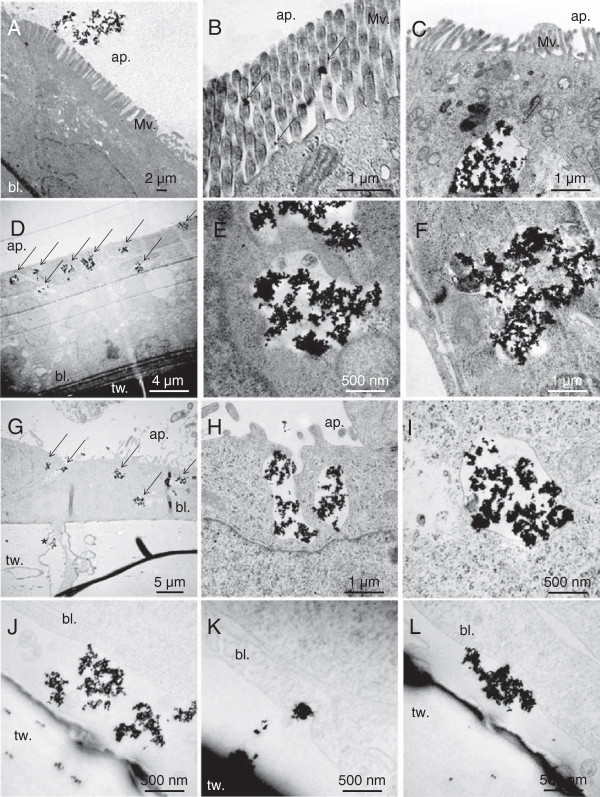
**TEM observation of TiO**_**2**_**-NP accumulation, in vitro.** TiO_2_-NP distribution in a Caco-2 monoculture **(A-C)**, a Caco-2/HT29-MTX co-culture **(D-F)**, and a Caco-2/RajiB co-culture **(G-L)** exposed on their apical pole to 50 μg/mL of TiO_2_-NPs for 48 h. tw.: transwell insert on which cells are grown, making possible the identification of the basolateral pole (bl.), on the opposite side of the apical pole (ap.). Ti-rich zones are pointed out with arrows.

We qualitatively analysed TiO_2_-NP transepithelial passage by following their route through the cell cross sections by transmission electron microscopy (TEM) (Figure 6). In the Caco-2 model NPs were observed outside the cells (Figure 6A), or entrapped between cell microvilli (Figure 6B, arrows). We observed NPs inside some of the cells, as agglomerates entrapped in cytoplasmic vesicles in the first micrometres from the apical membrane (Figure 6C). We never observed complete translocation of NPs through the Caco-2 epithelium.

In the Caco-2/HT29-MTX co-culture, intracellular agglomerates of NPs were more frequently observed (Figure 6D, arrows). NPs were mostly taken up by cells lacking microvilli, i.e. the HT29-MTX cells of the co-culture (Figure 6D). NPs were entrapped in cytoplasmic vesicles with diameters 250 nm-2 μm, located close to the apical membrane (Figure 6E-F). We never observed any NPs between the basolateral pole of cells and the transwell membrane.

Finally, in the Caco-2/RajiB co-culture, we observed intracytoplasmic vesicles filled with NPs (Figure 6G, arrows, Figure 6H-I). These vesicles did not show any specific location, they were distributed all across the cell layer (Figure 6G, arrows). Again, NPs were entrapped in cytoplasmic vesicles resembling endosomes (Figure 6H). Some agglomerates of NP were observed between the basolateral cell membrane and the surface of the transwell (Figure 6J-L). The basolateral cell membrane showed local invaginations, suggesting that NPs have been released from cells by exocytosis (Figure 6K-L). Note that in this model we also observed some M-cells (recognized because they have no microvilli, contrary to Caco-2 cells) crossing the transwell membrane and locating upside down on the membrane, in the basolateral compartment of the insert (Figure 6G, cross, and Additional file 7). We observed this phenomenon both in NP-exposed cells and in unexposed cells.

We then tried to quantify TiO_2_-NP translocation through these cell models by ICP-MS. When probing NP passage through control transwells, without any cells grown on its surface, Ti was retained in the basolateral compartment of 0.4 μm pore-sized transwells while they passed through 3 μm pore-sized transwells (not shown). This may be due to the agglomerated state of TiO_2_-NPs. Caco-2 and Caco-2/HT29-MTX models were grown on 0.4 μm pore-sized transwells while the Caco-2/RajiB model was grown on 3 μm pore-sized transwells. Consequently although ICP-MS is generally a valuable method for assessing NP translocation through epithelia, it was not a relevant method in the experimental conditions that we used.

## Discussion

In vivo and ex vivo, we show that agglomerates of TiO_2_-NPs are translocated through both the regular gut epithelium and through the FAE. Still TiO_2_-NP transepithelial passage is low, as previously reported by others after exposure of rats to dispersed TiO_2_-NPs [13]. The amount of TiO_2_-NPs accumulated in gut tissues is so low that we were unable to quantify it by PIXE (local limit of detection: 20-30 ppm). These NPs cause ex vivo and in vivo increase of paracellular permeability, which is correlated to down-regulation of the expression of genes encoding junction proteins. NPs may thus translocate through the regular ileum via impaired paracellular junctions.

Significant gut absorption of Ti and accumulation in the internal organs is observed in the studies by Zhao et al., Gui et al. and Cui et al. [9,11,12]. This discrepancy can be related to the different NP dispersion state and animal administration procedures. In the study by Cho et al. and in our work, NPs are prepared in water or in reconstituted gastric fluid, they are dispersed as suspensions with hydrodynamic diameter lower than 100 nm and administered to animals by gavage [13]. In the studies by Zhao et al., Gui et al. and Cui et al., TiO_2_-NPs are suspended in hydroxypropylmethylcellulose (HPMC); their hydrodynamic diameter is 208-300 nm; the animals are exposed by intragastric administration. The surface coating, hydrodynamic diameter may thus be critical parameters affecting TiO_2_-NP absorption through the gut.

In vitro, we show that agglomerates of TiO_2_-NPs pass only through the model of FAE, possibly by transcellular transport. The Caco-2/RajiB model has already been reported as allowing latex beads [26,27], polystyrene beads [28] and Ag-NPs and/or ions [29] to be translocated. We provide here evidence that TiO_2_-NPs, i.e. NPs that may be present in food products, may also translocate through this model of FAE. In the in vitro models of regular ileum epithelium that we used, TiO_2_-NPs neither cross the epithelium via the transcellular route nor via the paracellular route. We do not observe any increase in permeability or cell leakiness. Still the expression of genes encoding cell junction proteins is modulated, suggesting a remodelling of cell junctions. Impairment of cell junctions by TiO_2_-NPs has already been described after chronic exposure of Caco-2 enterocytes to a commercial suspension of NPs prepared in serum free cell culture medium [25]. Such exposure leads to a loss of epithelial integrity, with microvilli disorganization and subsequent TiO_2_-NP transepithelial translocation [25]. It was also demonstrated by Mahler et al. in a tri-culture gut model exposed to 50 nm and 200 nm polystyrene beads [30]. NP-induced cell junction impairment has also been described on models of the endothelial barrier. An invoked mechanism is activation of Akt due to oxidative stress, which further inactivates GSK-3β. GSK-3β then induces microtubule remodelling which increases permeability and causes cell leakiness [42]. A novel mechanism of cell junction impairment was recently reported, also on endothelial cells [43]: permeability increases before the onset of oxidative stress and independently of NP intracellular accumulation. The authors demonstrate that due to their small size, NPs migrate into the adherens junctions and directly bind to VE-cadherin, inducing its dephosphorylation which further abolishes its interaction with p120 and catenin. While interacting with VE-cadherin, TiO_2_-NPs also trigger actin remodelling via the activation of VE-cadherin pathway, resulting in endothelial cell leakiness (ECL). This novel non-receptor binding mechanism of ECL is called nanoEL [43]; it results from intracellular processes similar to those induced by histamine or VEGF, but occurs independently of a classical receptor-ligand interaction [43]. Due to their agglomerated state, the TiO_2_-NPs that we used probably did not gain access to adherens junctions, and consequently could not induce nanoEL.

Our ex vivo/in vivo and in vitro results related to NP-induced paracellular junction disruption are thus contradictory. Disruption is observed in the in vivo and ex vivo models while it is not observed in the in vitro models. The expression of the gene encoding ZO-1 is upregulated in vitro while it is down regulated in vivo. This discrepancy cannot be explained by different surface properties of TiO_2_-NPs, which would be caused by their preparation in different exposure media. Indeed their surface charge, coating with proteins and agglomeration state are similar. Our hypothesis is that the ex vivo and in vivo gut tissues are more sensitive to TiO_2_-NPs than the in vitro models that we used. Moreover, ex vivo and in vivo gut tissues were exposed to TiO_2_-NPs for 2 h and 6 h, while in vitro models were exposed for 6-48 h. Consequently another hypothesis is that cell junctions are also impaired in vitro, but that they are actively repaired by de novo expression of TJ and AJ proteins, as suggested by our in vitro gene expression results. We observe an early drop in claudin expression both in vitro and in vivo, that may interpreted as a first stage of junction disruption. This drop can be a consequence of oxidative stress, which has been shown to reduce the expression of tight junction proteins and among them claudin [44] as well as to increase paracellular permeability in gut and endothelial cells [42,44]. Oxidative stress is a canonical mechanism of TiO_2_-NP-induced toxicity [45] and we previously showed that the TiO_2_-NPs that we used here induced an early ROS production in lung cells [46]. In lung cells, intracellular ROS content continuously increases from 15 min to 4 h of exposure and then remains stable. If this kinetics is also true in gut cells, it can explain the early reduction of claudin expression which then returns to the basal level after 48 h of exposure. This drop in claudin expression may be sensed by cells as a stress signal. Cells would then respond by inducing de novo expression of junctional proteins, and among them catenin and ZO-1, in a view to reinforce tight and adherens junctions.

We observe low accumulation of TiO_2_-NPs and their sequestration in the most apical cytoplasm of the Caco-2 model. Conversely surface-treated TiO_2_-NPs, composed of a core of rutile coated with a layer of aluminum hydroxide and surface-treated with polydimethylsiloxane (PDMS), do not accumulate in Caco-2 cells [24]. Upon dilution in aqueous media the PDMS layer is removed from the surface of these NPs, still the aluminum hydroxide coating remains. Consequently the surface characteristics of these surface-treated NPs are certainly very different from the surface characteristics of the TiO_2_-NPs that we used, particularly their protein corona. This may explain that they are not accumulated in Caco-2 cells, while bare TiO_2_-NPs are. In the Caco-2/HT29-MTX coculture, TiO_2_-NPs are principally accumulated in the HT29-MTX cells. This is not surprising since Goblet cells are still able to undergo endocytosis while mature enterocytes are not [21]. The size and distribution of Ti-rich regions that we observe inside cells suggests intracellular accumulation via macropinocytosis or phagocytosis, rather macropinocytosis since Goblet cells are not phagocytosis-competent [47,48]. Accumulation of NPs in cell cytoplasm, entrapped in vesicles resembling lysosomes, may result in lysosome dysfunction. Lysosome dysfunction is known to cause dysregulation of autophagy, which is emerging as a general mechanism of NP-induced toxicity [49]. Among the recent literature related to this phenomenon, the most biopersistent NPs such as TiO_2_-NPs [50] or silica NPs [51] have been shown to induce autophagy and to block the flux of autophagosomes and fusion of autophagosomes with lysosomes in a variety of cell lines. As a consequence, damaged organelles including mitochondria accumulate in NP-exposed cells, leading to ROS production, oxidative stress, inflammation, DNA damage and eventually apoptosis [49]. We do not observe overt cytotoxicity and apoptosis in TiO_2_-NP exposed cells, but the altered gene expression that we observe could be an indirect consequence of autophagy dysregulation, through ROS intracellular production. In the Caco-2/RajiB co-culture, NPs are accumulated in vesicles distributed in the whole cell cytoplasm. Since M-cells are specialized in macromolecule transcytosis, it is possible that these vesicles are transcytosis vesicles. We never observed NP accumulation in cell nuclei, contrary to what happens for instance in buccal cells exposed to SiO_2_-NPs [52]. Finally we provide direct evidence that anatase, 12 nm TiO_2_-NP are not dissolved when they are stored in Caco-2 cells, and would consequently persist in gut cells where they accumulate, possibly leading to long-term impact.

Regarding TiO_2_-NP gut toxicity, two recent studies show that TiO_2_-NPs exert toxic effects on undifferentiated Caco-2 cells when they are prepared in serum-free medium. They induce cell mortality associated with the surface-area dose and the anatase/rutile ratio [22,23]. We do not observe any cytotoxicity. This can be explained by the surface characteristics of TiO_2_-NPs: the NPs that we used were prepared in serum-containing medium, i.e. are coated with proteins, agglomerated and negatively-charged. The interaction of NPs with cell membranes is governed by their surface properties. Negatively-charged NPs would be prone to electrostatic repulsion from the negatively-charged cell membrane, the interaction of NPs with cell membrane is thus restricted to specific recognition of membrane receptors by the proteins that coat their surface [53,54]. Conversely when prepared in serum-free exposure medium, NPs strongly adhere to the cell membrane and are non-specifically accumulated. Consequently, the impact of NPs is lower when prepared in serum-containing medium, as already shown for Au-NPs, polystyrene-NPs and SiO_2_-NPs [53,55].

## Conclusions

Ex vivo and in vivo we show that, upon acute exposure, TiO_2_-NP pass through both the follicle-associated epithelium and the regular intestinal epithelium and localize in the tissues below these epithelial layers. This can be considered as the first stage of translocation. TiO_2_-NP exposure results in an increase of paracellular permeability, certainly via the disruption of cell junctions. In vitro, we observe transcellular transport of TiO_2_-NPs only through the model of FAE. TiO_2_-NP accumulation highly depends on the cell model, being much higher in Goblet cells and M-cells than in enterocytes. At the gene expression level, TiO_2_-NP accumulation induces a deregulation of genes encoding proteins involved in epithelial structure maintenance. It suggests possible paracellular translocation of NPs through disrupted junctions if they are not appropriately repaired. We finally show that TiO_2_-NPs do not dissolve upon sequestration in gut cells, suggesting that they would possibly persist in the gut epithelium. Note that this study was done with high concentrations of NPs, mimicking a worst-case scenario, and now needs to be completed with data obtained on the same cell models exposed chronically to lower concentrations of TiO_2_-NPs.

## Methods

### Nanoparticle synthesis, dispersion and physico-chemical characterization

The TiO_2_-NPs we used were produced in our laboratories [56]. Their specific surface area, crystalline phase and grain size were measured by the Brunauer, Emmett and Teller (BET) method, X-ray diffraction and TEM, respectively, as described previously [46,57]. NPs suspensions (10 mg/mL) were prepared in ultrapure sterile water by high-power pulsed sonication (Vibra Cell 75043, 20 kHz, Bioblock scientific, 28% amplitude, 1s on/1s off, 4°C) using a 13 mm probe. The power of our sonicator was measured using the calorimetric procedure [58]. Amplitude of 28% corresponds to 16.7 W (immersion depth of the 3 mm microtip: 10 cm). NP agglomeration state was followed by dynamic light scattering (DLS, Malvern ZetaSizer 3000HS) and TEM observation; zeta potential was measured on the same equipment. This was carried out on NP suspensions diluted in DMEM supplemented with 10% (v/v) FBS, Ringer solution supplemented with 10% (v/v) FBS, 2.35 g/L pepsin prepared in 0.1N HCl (modelled gastric fluid) or 2.35 g/L pepsin prepared in 0.1N HCl to which was added pancreatin and bile extract (modelled intestinal fluid). DLS measurements were carried out on four independent samples in each condition, TEM measurements were carried out on 10 TEM grids and at least 50 NP agglomerates per TEM grid were counted. For grid preparation, a droplet of NP suspension was deposited on the grid and removed by aspiration after 5 min. The grid was then rinsed 3 times with ultrapure water and dried. TiO_2_-NPs, 5 nm, prepared in our laboratories and Evonik P25 were used as reference for XAS analyses.

### In vitro cell culture models

Caco-2 (ATCC HTB-37, passages from 39 to 45), Caco-2 clone 1 and HT29-MTX cells (provided by F. Barreau and T. Lesuffleur, respectively, INSERM U843 and U938, Paris) were grown in DMEM containing 10% (v/v) FBS, 2 mM L-glutamine, 1% (v/v) non-essential amino acids, 50 UI/mL penicillin and 50 μg/mL streptomycin. Cells were grown at 37°C in a 5% CO_2_ humidified atmosphere incubator. Caco-2 cells were cultured to 21 days post-confluence on Transwell-Clear® membranes (polyester, 0.4 μm pores, Costar) for complete differentiation. To reproduce a mucus-secreting epithelium, Caco-2 cells were co-cultured with HT29-MTX cells at the ratio of 75%/25% (Caco-2/HT29-MTX) for 4 weeks. Mucus secretion was probed by staining with 1% Alcian blue for 1 h [34]. To reproduce an in vitro FAE, Caco-2 clone 1 cells were grown on the upper side of transwell membranes (polyester, 3 μm pores, Costar). After fourteen days, RajiB lymphocytes (ATCC CCL-86) grown in RPMI supplemented with 10% FBS, 1% non-essential aminoacids, 0.01% β-mercaptoethanol, 50 UI/mL penicillin and 50 μg/mL streptomycin, were added in the basolateral compartment.

For the immunostaining of tight junction proteins, cells were fixed in 3% paraformaldehyde, permeabilized with 0.2% Triton X-100. They were incubated with rat anti-TJP1 antibody (Chemicon Int., 1:100 vol/vol), then with anti-rat FITC antibody (Sigma 1:200 vol/vol), washed and stained with 0.1 μg.mL^-1^ 4',6-diamidino-2-phenylindole (DAPI). Observations were performed with a fluorescence microscope (Zeiss). ALP activity was measured using BioVision kit (Moutain View, USA). The kit uses *p-*nitrophenylphosphate as a phosphatase substrate which turns yellow (λmax = 405 nm) when dephosphorylated by ALP. ALP concentrations were then determined by comparison to a calibration curve. Fast red staining was performed as described by the supplier (Sigma-Aldrich), i.e. 1 pellet of Fast red and 1 pellet of Tris were dissolved in 1 mL of ultrapure water; this solution was applied to the cell monolayer for 10 min at 27°C. In the absence of specific markers of human M-cells, differentiation of Caco-2 cells into M-cells was confirmed by TEM observation and measurement of the expression of ALP, sucrase isomaltase (SI), claudin 5 and 8 (CLDN5, CLDN8), occludin (OCCL) and myosin light chain kinase long isoform (MLCK).

### Ex vivo and in vivo models

In the ex vivo experiment, Peyer’s patches and regular ileum from mice were dissected and immediately mounted in Ussing chambers [35], then exposed to 50 μg/ml NPs prepared in Ringer solution to which 10% (v/v) FBS was added. This suspension was continuously circulated in the Ussing chamber for 2 h, under oxygenation. Ileum and Peyer’s patches were then rinsed and prepared for imaging experiments. In the in vivo experiment, mice were exposed by a single oral gavage to 12.5 mg/kg of TiO_2_-NPs dispersed in 150 μl of water. After 6 h, corresponding to a digestion cycle, mice were sacrificed; portions of ileum epithelium and Peyer’s patches were sampled and prepared for imaging experiments.

### Imaging and quantification

#### Transmission electron microscopy

**Figure 7:**
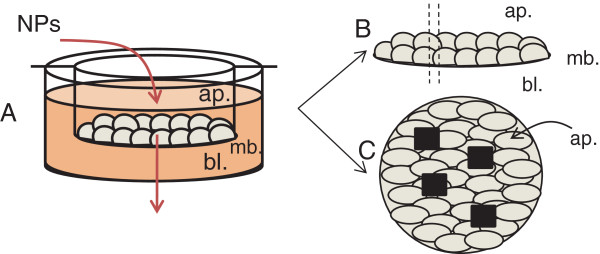
**Exposure protocol.** Gut cells were grown on semi-permeable transwell inserts then exposed to 50 μg/mL of TiO_2_-NPs on their apical pole **(A)**. Cells were chemically fixed, embedded in Epon resin and cross sectioned for TEM observation and SR-μXRF imaging **(B)**; or transwell membranes were dissected and deposited on an appropriate sample holder, taking care of preserving epithelial polarity, cryofixed, freeze-dried and analysed for their NP accumulation through global μPIXE/μRBS analysis of the cell monolayer (4 areas of 50 μm×50 μm) **(C)**.

After apical exposure to NPs (Figure 7A), transwell membranes were dissected, rinsed with PBS, fixed in 2% glutaraldehyde in cacodylate buffer and in 1% osmium tetroxide solution. They were dehydrated through a graded series of ethanol and embedded in Epon resin, taking care of preserving their polarity. Ultra-thin sections were cut and stained for 10 min with 1% uranyl acetate for observation in transmission electronic microscopy (TEM) with a Philips CM120 electron microscope operating at 80 kV (Figure 7B).

#### Micro X-ray fluorescence imaging and X-ray absorption spectroscopy analysis

Samples embedded for TEM observation were also analysed by SR-μXRF and μXAS. Three μm cross-sections were cut and sandwiched between 4 μm thick Ultralene® foils (SPEX SamplePrep). SR-μXRF was used to map trace element distribution (Ti, K, Ca, P/Os and Cl); it detects concentrations down to a few ppm. Experiments were carried out at the LUCIA (SOLEIL, France) [59] and ID21 (ESRF, Grenoble, France) Beamlines. Images presented in this article are from ID21 beamline. The X-ray beam was focussed to 0.2 × 0.8 μm^2^ (V × H) by means of a Tungsten Fresnel Zone Plate lens. Maps were acquired at the fixed energy of 5.1 keV, with a 1 × 1 μm^2^ step, and data were processed using PyMCA [60]. Cells were identified by mapping K or P/Os distribution. TiO_2_-NP intracellular distribution was mapped through Ti-distribution imaging. XAS analyses were performed on cell regions containing high amounts of Ti, directly after SR-μXRF images acquisition on ID21. XAS spectra were registered at the Ti K-edge (4.966 keV, energy scan between 4.94 and 5.10 keV, energy step 0.25 eV), background subtracted and normalized by their post-edge linear trend using Athena [61] as described previously [39]. The pre-edge region (4.972 to 4.985 keV) was fitted using Fityk [62] by deconvolution using an arctangent and four Gaussian functions [40,41].

#### Micro particle-induced X-ray emission

For μPIXE and micro Rutherford Backscattering, transwell membranes were rinsed with PBS cryofixed by immersion in isopentane chilled to -160°C in liquid nitrogen. Samples were freeze-dried for 24 h at -10°C, 0.37 mbar (Figure 7C). Micro-PIXE and μRBS spectra were recorded simultaneously on the nanobeam line of the AIFIRA platform (CENBG, Bordeaux, France) [63]. The 3.5 MV Singletron accelerator (HVEE) was adjusted in order to deliver a focused beam (2.5 μm) of 3 MeV protons, with a beam current of 1 nA. X-rays were detected with a 80 mm^2^ Si(Li) detector (Gresham, energy resolution: 160 keV) orientated at 135° with respect to the incident beam axis, and equipped with a 12 μm thick beryllium window. A funny filter (Al, thickness 200 μm, % hole = 1 mm) was used in order to limit the dead-time (Rate ~ 500 cps/s, dead time below 10%). Backscattered protons were recorded at 135° with a silicon PIPS detector (Canberra, 25 mm^2^, thickness 100 μm, resolution: 17 keV). Four elemental maps of 50×50 μm^2^ were recorded on each sample, and drawn using the SupaVISIO software (http://barbotteau.software.informer.com/). For Ti intracellular content measurement, data were fitted using SIMNRA (RBS) [64] and Gupix (X-ray spectra) [65] making possible the semi-quantification of Ti/P content. The Si signal probably rises from the emission of the Si crystal of the detector.

### Assessment of the impact of NPs

#### TEER measurements

Millicell-ERS (Millipore) was used to measure the TEER. TEER was calculated as follows: TEER = (R_cell_ ‒ R_blank_) × A, where TEER is expressed in Ω.cm^2^, cell and blank resistances in Ω and A, the surface area of the insert, in cm^2^.

#### Cytotoxicity assays

Cells grown on transwell inserts were exposed to 0 to 500 μg/mL of NPs up to 48 h. Reduction of cell metabolic activity, reflecting NPs cytotoxicity, was assessed by using 3-(4,5-dimethylthiazol-z-yl)-2,5-diphenyl-tetrazotium bromide (MTT). After exposure, medium was replaced by 0.5 mg/mL MTT, then after 2 h at 37°C formazan crystals were dissolved in DMSO. Plates were then centrifuged allowing NPs to sediment. Supernatants were transferred into another plate for absorbance measurements at 550 nm [57]. This procedure ensured that TiO_2_-NPs did not interfere with the absorbance reading. Results of interference assessment are shown in Additional file 4. For trypan blue exclusion assay, cells were trypsinized and stained for 5 min with trypan blue (v/v). Blue and white cells were counted. For apoptosis detection, cells were stained with a mixture of acridin orange (70 μg/mL) and ethidium bromide (150 μg/mL) for 5 min as described by Mironova et al. [66]. They were deposited on a glass slide and observed on a fluorescence microscope with excitation at 470 nm and emission at 517 nm. Acridin orange stains double-strand nucleic acid in green, but is also trapped in acidic compartments, resulting in an orange-red fluorescence. The nuclei of apoptotic cells undergo a series of transformations and among them they acidify; acridin orange thus stains the nuclei of apoptotic cells bright yellow-orange. Ethidium bromide is taken up in necrotic cells only and stains red double-strand DNA. Consequently upon labelling with acridin orange/ethidium, live cells look green with a punctated red cytoplasmic (lysosomes). Necrotic cells are homogeneously stained in red. The cytoplasm of apoptotic cells looks green with punctated red staining, while their nuclei look yellow-orange.

#### Transport experiments

Paracellular permeability was assessed by measurement of mucosal-to-serosal flux of Dextran-FITC 4 kDa as described earlier [67]. Dextran-FITC was added in the NP suspension immediately before exposure and FITC fluorescence was measured during 2 h in the serosal compartment of the Ussing chamber (ex vivo experiment), after 6 h in the bloodstream (in vivo experiment). In vitro, after 48 h of exposure to NPs, 10^-5^ M of Dextran-FITC 4 kDa was added in the apical compartment of the transwell. FITC fluorescence was monitored in the basolateral compartment after 1 h and 2 h. In vitro, transport experiments were also performed with radiolabelled molecules; which are more sensitive than Dextran-FITC 4 kDa flux measurements. After exposure to NPs transwells were rinsed three times with PBS, the apical and basolateral media were replaced by transport buffer (150 mM NaCl, 5.2 mM KCl, 2.2 mM CaCl_2_, 0.2 mM MgCl_2_, 6 mM NaHCO_3_, 2.8 mM glucose and 5 mM Hepes). [^14^C]-Sucrose (0.1 mCi.mL^-1^) and [^3^H]-vinblastine (250 μCi.mL^-1^) (Amersham, Buckinghamshire, UK) were added in the apical compartment (either apical or basolateral). After 60 min, ^3^H concentration was determined by scintillation counting in the apical and basolateral compartments. The permeability coefficient P_app_ value was calculated as P_app_ = (dQ/dt)/(A × C_0_), where dQ/dT is the amount of compound transported per unit of time, A is the membrane surface area and C_0_ is the initial apical concentration. The mass balance (R) was calculated as R (*%*) = 100 × [(A + D)/D_0_], where A and D are the amount of radiolabelled molecule in the basolateral and apical compartment, respectively, and D_0_ is the amount of radiolabelled molecule introduced at t = 0. The mass balance was always between 80 and 120%.

#### Real-time semi-quantitative polymerase chain reaction (RT-qPCR)

RNA was extracted using GenElute^TM^ mammalian total RNA kit (Sigma Aldrich) and reverse-transcribed with random primers using the RT^2^ first strand kit (Superarray Bioscience Corporation, Frederick, USA). Each primer set was used at 0.4 μM in a specific RT2 profiler PCR array according to the manufacturer’s instructions. Relative expression values were calculated as 2^-ΔΔCt^, where ΔCt is the difference between the amplification curve (Ct) values for genes of interest and the housekeeping gene (glyceraldehyde 3-phosphate dehydrogenase, GAPDH). Expression level was considered too low to be reliable when Ct was higher than 33 [68].

### Statistical analyses

Experiments were repeated independently three times. Non-parametric one-way analysis of variance on ranks approach (Kruskal-Wallis) was performed using the Statistica 7.1 software (Statsoft, Chicago, USA). Paired comparisons were run using Mann-Whitney u-tests and results were considered statistically significant (*) when p < 0.05 or (**) when p < 0.01.

## Abbreviations

NP: Nanoparticle; FAE: Follicle-associated epithelium; XRF: X-ray fluorescence; XAS: X-ray absorption spectroscopy; PIXE: Particle-induced X-ray emission; TEM: Transmission electron microscopy; DLS: Dynamic light scattering; PdI: Polydispersity index; MTT: TEER, transepithelial resistance; ICP-MS: Inductively-coupled plasma-mass spectrometry.

## Competing interests

The authors declare that they have no competing interests.

## Authors’ contributions

EB carried out cell culture, toxicity and transport assays, electron microscopy preparations and observations, as well as sample preparation for X-ray absorption, X-ray fluorescence and PIXE experiments. GV and BF carried out the X-ray absorption and X-ray fluorescence experiments. FB carried out the in vivo and ex vivo experiments, cultured and characterized the Caco-2/RajiB co-culture. CC and NH produced and characterized the nanoparticles. SS carried out the PIXE experiments. CCA and TR carried out the proteomic analyses of nanoparticle protein corona. AM participated in qPCR and transport experiments and in the conception of this study. MC conceived, designed and coordinated the study, and drafted the manuscript. All authors read and approved the final manuscript.

## Supplementary Material

Additional file 1 **TEM images and size distribution of TiO**_**2**_**-NPs.** Two TEM images show NP suspensions in water and in cDMEM, and a histogram describes the size distribution (DLS) of these suspensions. Click here for file

Additional file 2 **Composition of the protein corona on TiO**_**2 **_**nanoparticles.** The protein corona of TiO_2_-NPs in cDMEM and Ringer + FBS is provided in two tables, with the corresponding Method section. Click here for file

Additional file 3 Characterization of cell models. One table and three figures that describes the complete characterization of the three cell models. Click here for file

Additional file 4 **NP interference with MTT assay.** Interference is probed by addition of TiO_2_-NP to dissolved formazan crystals, and absorbance measurement. Click here for file

Additional file 5 **PIXE images of Ti accumulation in HT29-MTX monoculture cross-sections.** PIXE images of cell cross-sections, showing that TiO_2_-NPs are really accumulated in the cells, and not adsorbed on the cell membrane. Click here for file

Additional file 6 **Energy dispersive imaging and electron diffraction analyses of a TiO2-NP agglomerate inside a M-cell.** This additional file shows TEM images and their EDS analysis, together with the electron diffractogram recorded on the electron-dense agglomerate of NPs located inside the M-cell and its analysis. Click here for file

Additional file 7 **M-cells crossing the transwell insert membrane.** Three TEM images show M-calls crossing the transwell membrane, along with the nanoparticles they contain. Click here for file
