# Supplementary material for: Titanium dioxide nanoparticle impact and translocation through ex vivo, in vivo and in vitro gut epithelia
Source: Part Fibre Toxicol. 2014 Mar 25;11:13. doi: 10.1186/1743-8977-11-13 (PMC3987106; doi:10.1186/1743-8977-11-13)
Supplement: Additional file 1 — TEM images and size distribution of TiO2-NPs. Two TEM images show NP suspensions in water and in cDMEM, and a histogram describes the size distribution (DLS) of these suspensions. [file 1743-8977-11-13-S1.pdf]

TEM images and size distribution of TiO_2-NPs

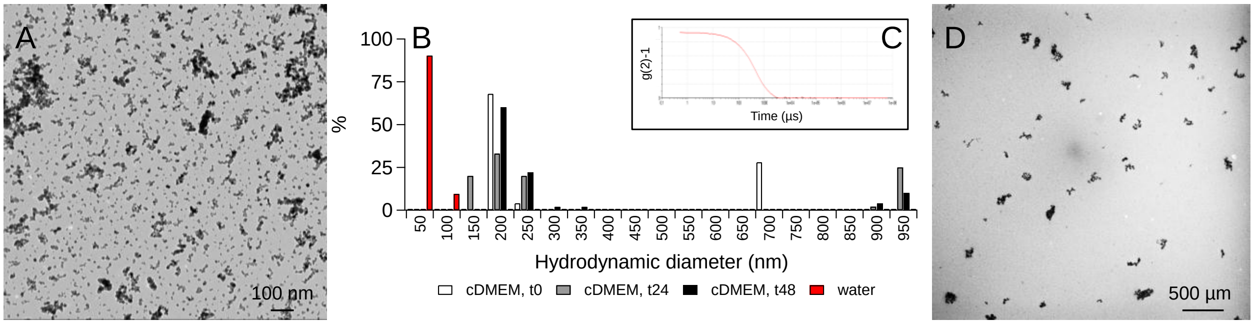

TEM images and size distribution of TiO_2-NPs. TEM images were recorded on TiO_2-NPs in water (A). NP size distribution was evaluated by PCS measurement after sonication in water (“water”) or sonication in water and dilution in cell culture medium containing 10% of SVF (“cDMEM”, for complete DMEM). Size range of NP agglomerates either immediately after dilution (“cDMEM, t0”), or 24 h (“cDMEM, t24”) or 48 h (“cDMEM, t48”) after dilution (B). Typical correlogram recorded during a PCS measurement (C). TEM images of TiO_2-NP agglomerates 24 h after dilution in exposure medium (D).
