# Supplementary material for: Titanium dioxide nanoparticle impact and translocation through ex vivo, in vivo and in vitro gut epithelia
Source: Part Fibre Toxicol. 2014 Mar 25;11:13. doi: 10.1186/1743-8977-11-13 (PMC3987106; doi:10.1186/1743-8977-11-13)
Supplement: Additional file 2 — Composition of the protein corona on TiO2 nanoparticles. The protein corona of TiO2-NPs in cDMEM and Ringer + FBS is provided in two tables, with the corresponding Method section. [file 1743-8977-11-13-S2.pdf]

Composition of the protein corona on TiO_2 nanoparticles

Table 1 (1/2). Composition of the protein corona on TiO_2-NPs in cDMEM[a]

Protein name	Protein accession numbers	nr. unique pept.	nr. unique spectra	nr. total spectra	% sequ. cov.
Complement C3	sp|Q2UVX4	48	64	69	35.6%
Serum albumin	sp|P02769	26	36	48	41.7%
probable Complement component 4	tr|E1BH06	24	30	30	19.7%
Apolipoprotein A-I	sp|P15497	16	25	32	60.4%
Hemoglobin fetal subunit beta	sp|P02081	11	17	23	84.8%
Alpha-2-HS-glycoprotein	sp|P12763,tr|B0JYN6	11	18	25	44.8%
Antithrombin-III	tr|F1MSZ6	11	12	13	31.4%
Serotransferrin	sp|Q29443,tr|G3X6N3	10	11	11	17.5%
Complement component C9	sp|Q3MHN2	9	9	10	18.8%
Thrombospondin-1	sp|Q28178	7	7	7	7.18%
Alpha-fetoprotein	sp|Q3SZ57	7	8	8	14.9%
Pigment epithelium-derived factor	sp|Q95121	7	8	8	27.4%
Coagulation factor V	sp|Q28107	5	5	5	2.67%
Hemoglobin subunit alpha	sp|P01966	5	5	6	2.94%
Alpha-1-antiproteinase	sp|P34955	5	5	5	16.1%
Collagen alpha-1(I) chain	sp|P02453	4	5	6	2.94%
Prothrombin	sp|P00735	4	4	4	8.00%
Apolipoprotein E	sp|Q03247,tr|Q0ZCB4	4	5	5	16.5%
Kininogen-1	sp|P01044	4	4	4	8.53%
Complement Factor I	tr|F1N4M7,tr|Q32PI4	4	7	7	8.25%
Collagen alpha-2(I) chain	sp|P02465	3	4	4	2.71%
Tetranectin	sp|Q2KIS7	3	5	5	22.8%
probable Complement component 4	tr|F1MVK1	3	3	3	11.1%
SERPING1 protein	tr|E1BMJ0	3	3	3	11.1%
Complement C5a anaphylatoxin	tr|F1MY85	3	3	3	2.44%

Table 1 (2/2). Composition of the protein corona on TiO_2-NPs in cDMEM[a]

Protein name	Protein accession nr.	nr. unique pept.	nr. unique spectra	nr. total spectra	% sequ. cov.
Insulin-like growth factor-binding protein	sp|P13384	3	3	3	8.83%
Vitamin D-binding protein	sp|Q3MHN5	3	3	3	6.96%
Adiponectin	sp|Q3Y5Z3	3	5	5	15.4%
Alpha-enolase	sp|Q9XSJ4,tr|F1MB08	3	4	4	8.76%
ALB protein	tr|B0JYQ0	3	6	6	41.7%
Complement Factor I	Q32PI4	3	6	6	6.15%
Coagulation factor XIII, B polypeptide	Q2TBQ1	3	3	3	7.11%
Coagulation factor XI	sp|Q5NTB3,tr|F1MUT4,tr|Q6PZ62,tr|Q6Q0I7	2	2	2	3.52%
putative tropomyosin alpha	tr|A6QR15	2	3	3	3.87%
Histidine-rich glycoprotein	tr|F1MKS5,tr|Q9BGU1	2	3	3	5.22%
Collagen alpha-1(XI) chain	tr|F1N0K0	2	2	2	2.37%
Actin, cytoplasmic 1	sp|P60712,sp|P63258,tr|F1MRD0	2	2	2	6.93%
SERPIND1 protein	tr|A6QPP2	2	2	2	4.44%
Coagulation factor X	sp|P00743,tr|Q3MHW2	2	2	2	4.07%
Apolipoprotein A-II	sp|P81644	2	2	2	26.0%
Plasma kallikrein	sp|Q2KJ63	2	2	2	3.62%
Elongation factor 1-alpha	Q862F6	2	2	2	5.19%

[a]Protein corona was analyzed by nano LC/MS-MS, peptides were identified by database searching using Mascot™ algorithm, using all proteins annotated for Bos taurus (Taxonomy ID 9913) in UniprotKB, their decoy equivalents and common contaminants (UniProtKB release of 2013-05-01, 63412 target+decoy entries). Nr.: number; pept.: peptide; sequ.: sequence; cov.: coverage.

Table 2. Composition of the protein corona on TiO_2-NPs in Ringer+FBS

**Protein name**	**Protein accession nr.**	**nr. unique pept.**	**nr. unique spectra**	**nr. total spectra**	**% sequ. cov.**
Complement C3	sp|Q2UVX4	26	29	29	20.5%
ALB protein	tr|B0JYQ0	19	25	39	34.4%
Apolipoprotein A-I	sp|P15497	14	17	21	51.3%
Hemoglobin fetal subunit beta	sp|P02081	12	16	22	84.8%
Alpha-2-HS-glycoprotein	sp|P12763	12	16	23	51.0%
probable Complement component 4	tr|E1BH06	7	7	7	5.51%
Hemoglobin subunit alpha	sp|P01966	6	8	11	57.0%
Antithrombin-III	tr|F1MSZ6	6	6	6	14.0%
Serotransferrin	sp|Q29443	5	5	5	12.6%
Collagen alpha-2(I) chain	sp|P02465	3	3	3	2.93%
Thrombospondin-1	Q28194	3	3	3	2.65%
Tetranectin	sp|Q2KIS7	3	3	3	16.3%
Apolipoprotein E	sp|Q03247	3	3	3	12.7%
Pigment epithelium-derived factor	sp|Q95121	3	3	3	13.5%
Complement component C9	sp|Q3MHN2	3	3	3	5.84%
Apolipoprotein A-II	sp|P81644	2	2	2	26.0%
Collagen alpha-1(I) chain	sp|P02453	2	3	3	1.57%
Coagulation factor V	sp|Q28107	2	2	2	1.22%
Alpha-1-antiproteinase	sp|P34955	2	3	4	6.01%
Alpha-enolase	sp|Q9XSJ4	2	2	2	4.61%
Vitamin D-binding protein	tr|F1N5M2	2	3	3	5.06%
Fibrinogen alpha chain	sp|P02672	2	3	3	3.74%
Alpha-fetoprotein	sp|Q3SZ57	2	2	2	4.59%
Complement factor I	Q32PI4	2	2	2	3.88%
Serum albumin	sp|P02769	2	3	5	25.4%
Prothrombin	sp|P00735	2	2	2	3.84%

Method for protein corona identification

Protein corona identification

Protein elution and electrophoretic in gel concentration

One microgram of TiO_2-NP water-suspension was incubated for 1 h at 4°C in 2 mL of DMEM+10% FBS or Ringer solution +10% FBS, then washed three times for 5 min at 4°C with 1 mL of 10 mM HEPES buffer, on a rotating wheel. This washed TiO_2-NP pellet containing the adsorbed proteins was extracted for one hour on a rotating wheel with 125 µl of elution buffer (8M urea, 50mM DTT, 100mM Tris-HCl, PH 7.5). The suspension was then centrifuged at 15,000g for 20 minutes, the supernatant was saved and its protein concentration determined with a Bradford assay. SDS (2% final concentration) and tracking dyes (bromophenol blue and pyronin Y) were than added. Ten micrograms of proteins were loaded for each sample on a SDS gel (10 % acrylamide, 15x15x0.15 cm) on which a 5 cm long stacking gel was cast (5 % acrylamide in Tris HCl 125 mM pH 7.5). The samples were loaded on alternate lanes to avoid cross contamination, and the empty lanes were loaded with an equivalent amount of sample buffer, but using bromocresol green as the tracking dye. The gels were migrated at 50 V for 30 minutes and then at 100 V until they reached the middle of the stacking gel. The protein bands (blue band) were then excised, leaving as much as possible of free SDS (pink band) as possible.

In-gel digestions

After in-gel reduction and alkylation using the MassPrep Station (Waters, Milford, MA, USA), proteins were in-gel digested with trypsin at 37°C overnight. After digestion, peptides were extracted with a 60% acetonitrile, 0.1% formic acid solution prior to mass spectrometry analyses.

NanoLC-MS/MS analyses

Digestion peptides were analyzed on a nanoACQUITY UltraPerformance LC® (UPLC®) system coupled to a MaXis 4G Q-Tof (Bruker Daltonics, Bremen, Germany) equipped with a nano-spray ion source. The system was fully controlled by HyStar™ 3.2 (BrukerDaltonics). The peptides were first trapped on a nanoAcquityTM UPLCTM pre-column (Symmetry C18 Trap, 5 µm, 180 µm x 20 mm, Waters) and then separated on a nanoAcquity™ UPLC™ column (BEH C18, 1.7 µm, 75 µm x 250 mm, Waters). The solvent system consisted of 0.1% formic acid in water (solvent A) and 0.1% formic acid in acetonitrile (solvent B). Peptides were trapped during 1 min at 15 µL/min with 99 % A and 1 % B. Elution was performed at a flow rate of 450 nL.min-1, using a linear gradient of 6-35% B over 55 min at 45°C. The mass spectrometer was operating in positive mode, with the following settings: source temperature was set to 160°C while dry gas flow was at 5 liters per minute. The nanoelectrospray voltage was optimized to −5000 V. External mass calibration of the TOF was achieved using Tuning Mix (Agilent Technologies, Paolo Alto, USA) in the mass range of 300-2700 m/z. Mass correction was achieved by recalibration of acquired spectra to the applied lock masses (methylstearate ($[M+H]^+$ 299.2945 m/z) and hexakis(2,2,3,3,-tetrafluoropropoxy)phosphazine ($[M+H]^+$ 922.0098 m/z)). For tandem MS experiments, the system was operated with automatic switching between MS and MS/MS modes in the range of 100-2500 m/z (MS acquisition time of 0.4 s, MS/MS acquisition time between 0.05 s (intensity > 250000) and 1.25 s (intensity <5000)). The 6 most abundant ions (absolute intensity threshold of 1500), were selected from each MS spectrum for further isolation and CID fragmentation using nitrogen as collision gas. Peak lists in mascot generic format (.mgf)

were generated using DataAnalysis™ software (version 4.0; Bruker Daltonics, Bremen, Germany).

MS/MS data interpretation

Data from nanoLC-MS/MS analyses were interpreted by database searching using the Mascot™ algorithm installed on a local server (version 2.4.1, Matrix Science, London, UK). Spectra were searched against a database containing all proteins annotated for *Bos taurus* (Taxonomy ID 9913) in UniprotKB, their decoy equivalents and common contaminants (UniProtKB release of 2013-05-01, 63412 target+decoy entries). The database was built with our in-house developed software pipeline MSDA (Mass Spectrometry Data Analysis, https://msda.unistra.fr). Searches were performed with a mass tolerance of 10 ppm in MS and of 0.05 Da in MS/MS mode, allowing one missed cleavage. Carbamidomethylation of cysteine residues was set as fixed modification and oxidation of methionine residues and acetylation on protein N-termini were set as variable modifications. Following the guidelines for proteomic data publication [1,2] filtering criteria based on probability-based scoring of the identified peptides have been taken into account for high confidence identifications and false discovery rates in protein identification were controlled thanks to the decoy hits. The Scaffold™ software (v.3.6.5, Proteome software Inc., Portland, OR, USA) was used to validate identifications using the following criteria: proteins were validated when identified with a minimum of 2 unique peptides and MS/MS spectra showing ion scores higher than Mascot's threshold score of identity (95% confidence level) and ion scores higher then 25.

References

1. Carr S, Aebersold R, Baldwin M, Burlingame A, Clauser K, Nesvizhskii A: **The need for guidelines in publication of peptide and protein identification data : working group on publication guidelines for peptide and protein identification data.** *Mol Cell Proteomics* 2004, **3(6)**: 531-3.

2. Wilkins MR, Appel RD, Van Eyk JE, Chung MCM, Görg A, Hecker M, Huber LA, Langen H, Link AJ, Paik YK, Patterson SD, Pennington SR, Rabilloud T, Simpson RJ, Weiss W, Dunn MJ: **Guidelines for the next 10 years of proteomics.** *Proteomics* 2006, **6:** 4-8.
