# Supplementary material for: Titanium dioxide nanoparticle impact and translocation through ex vivo, in vivo and in vitro gut epithelia
Source: Part Fibre Toxicol. 2014 Mar 25;11:13. doi: 10.1186/1743-8977-11-13 (PMC3987106; doi:10.1186/1743-8977-11-13)
Supplement: Additional file 3 — Characterization of cell models. One table and three figures that describes the complete characterization of the three cell models. [file 1743-8977-11-13-S3.pdf]

**Characterization of the in vitro cell models**

Differentiated Caco-2 epithelium

Caco-2 cells were grown on Transwell-Clear® membranes (polyester, 0.4 µm pores, Costar). For complete differentiation into mature enterocytes they were maintained for 21 days after reaching confluence. Immunostaining of ZO-1 tight junction protein (Figure 1A) and TEM observation (Figure 1B) confirmed the presence of tight junctions; alkaline phosphatase activity (Figure 1C) and TEM observation (Figure 1D) confirmed the presence of a dense network of well-organized microvilli.

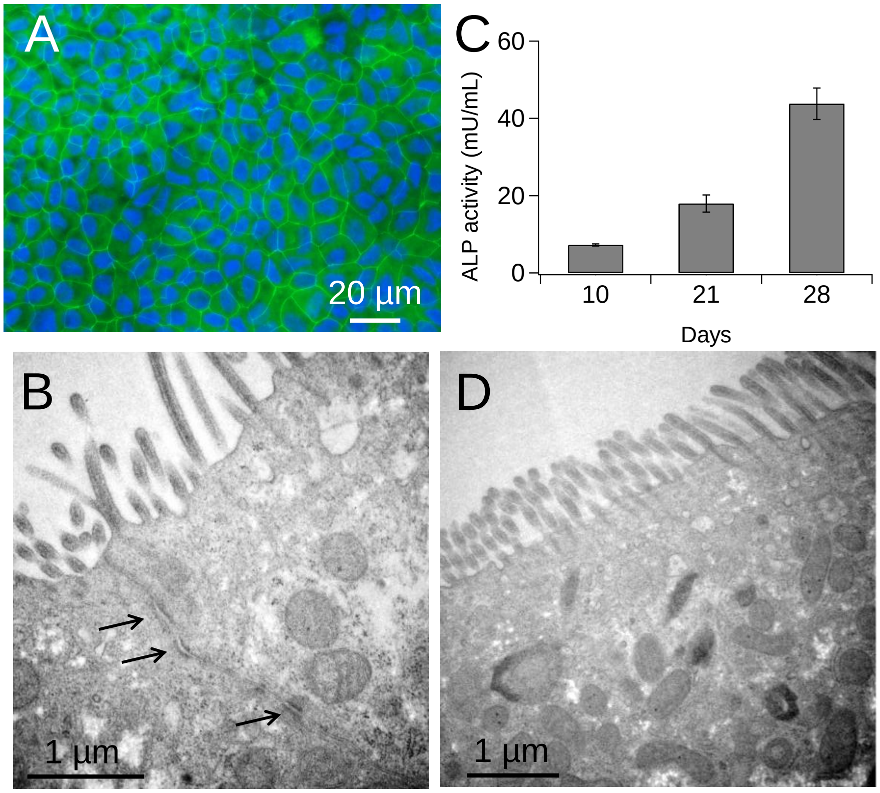


**Figure 1.** Differentiation of Caco-2 enterocytes. Complete differentiation was assessed by immunostaining of ZO-1 junction protein (A), TEM observation of the presence of tight junctions (B), their increased alkaline phosphatase activity (C), and TEM observation of well-organized microvilli on their apical surface (D).

Caco-2/HT29-MTX co-culture

To reproduce a mucus-secreting epithelium, Caco-2 cells were co-cultured for 4 weeks with HT29-MTX cells at the ratio of 75% Caco-2/25% HT29-MTX. Post-confluent HT29-MTX cells show a discrete brush border and secrete mucus [1]. Mucus secretion was probed by staining with 1% Alcian blue for 1 h (Figure 2A-C) [2]. HT29-MTX distribution in the co-culture was not homogeneous, and mucus production was restricted to the areas where HT29-MTX cells grew (intense blue staining in Figure 2A-C). The TEER (Figure 2D) and ALP activity (Figure 2E) were significantly lower in Caco-2/HT29-MTX co-culture than in the Caco-2 monoculture, proving that microvilli-containing cells were less abundant. Fast red staining of ALP (Figure 2F-H) showed areas in the Caco-2/HT29-MTX co-culture where ALP expression was reduced, confirming the presence of HT29-MTX cells.

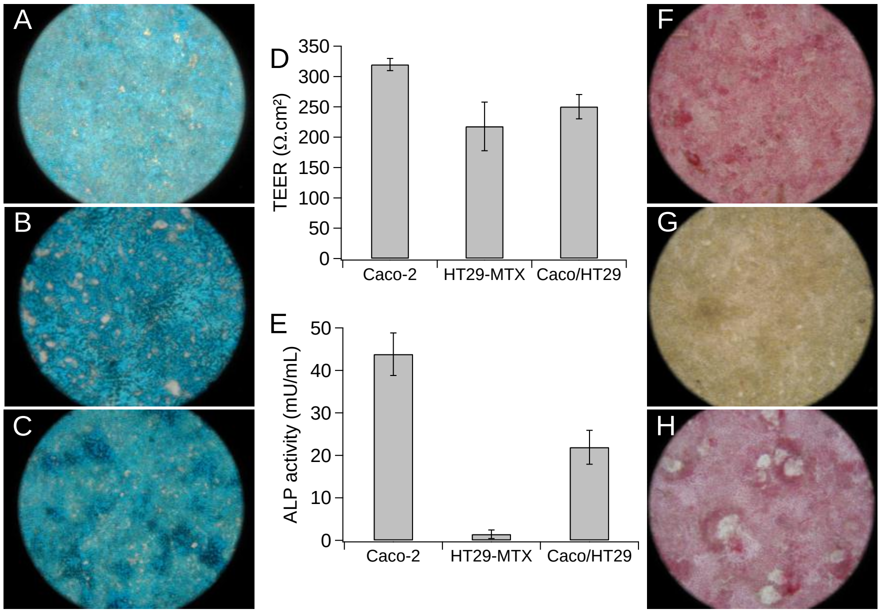


**Figure 2.** Characterization of the Caco-2/HT29-MTX coculture. Mucus staining in Caco-2 (A), HT29-MTX (B) and a coculture of Caco-2 and HT29-MTX (C) cells. Transepithelial resistance

(E) and alkaline phosphatase activity (F). Alkaline phosphatase staining using FastRed on Caco-2 (G), HT29-MTX (H) and a coculture of Caco-2 and HT29-MTX (I) cells.

Caco-2/RajiB co-culture

In the absence of specific markers of human M-cells, differentiation of Caco-2 cells into M-cells was confirmed by TEM observation (Figure 3). The epithelium was dense (Figure 3A), presented cell junctions and irregular microvilli (Figure 3B-C). ALP activity in Caco-2 clone 1 cells was 142 ± 13 mU/mL.min while it dropped to 71 ± 11 mU/mL.min in the Caco-2/Raji B co-culture.

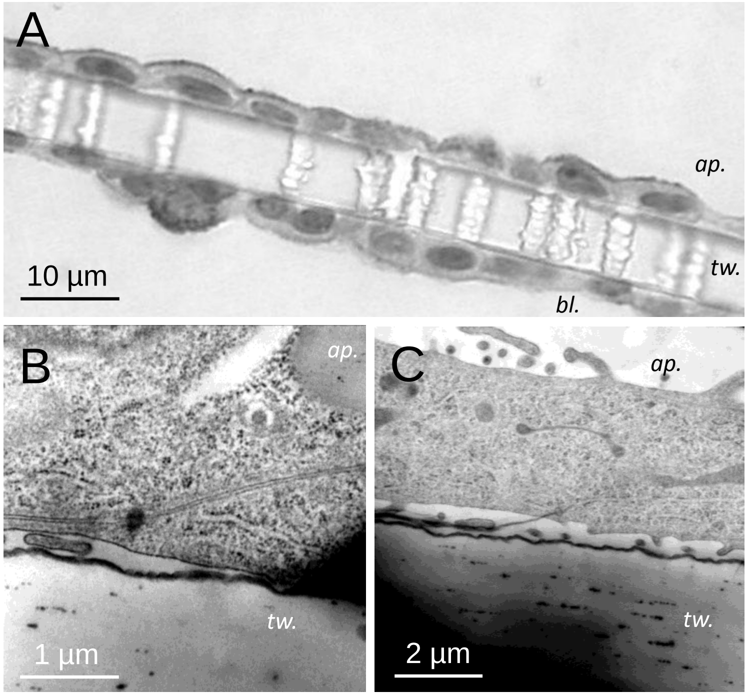


**Figure 3.** Microscopic observation of M-cells. A: optical microscopy image of the Caco-2/RajiB coculture, forming a dense and homogeneous epithelium. B, C: TEM images of Caco-2/RajiB coculture, presenting several features of M-cells: cell-cell junctions (B), disorganized and rare microvilli (C). ap.: apical compartment, bl. Basolateral compartment, tw.: transwell insert.

Reduced expression of ALP and sucrase isomaltase (SI), involved in tight junction structure, as well as claudin 5 and 8 (CLDN5, CLDN8), occludin (OCCL) and myosin light chain kinase long isoform (MLCK), involved in microvilli development, were observed (Table 1).

**Table 1.** Differential expression of genes involved in microvilli and tight junction structure[a]

| **Gene** | **ALP** | **SI** | **CLDN2** | **CLDN5** | **CLDN8** | **OCLN** | **TJP1** | **MLCK** |
|---|---|---|---|---|---|---|---|---|
| Caco-2 | 1.02±0.44 | 1.16±0.21 | 0.92±0.13 | 1.03±0.15 | 1.05±0.09 | 1.02±0.12 | 1.03±0.14 | 1.05±0.33 |
| Caco-2/RajiB | 0.13±0.05* | 0.38±0.21 | 0.91±0.32 | 0.54±0.12* | 0.62±0.24* | 0.59±0.19* | 0.95±0.12 | 1.91±0.60* |

[a]Differential expression was measured by RT-qPCR in Caco-2 monoculture and Caco-2/RajiB coculture. Expression is expressed as fold increase ($2^{-\Delta\Delta Ct}$) where Ct is the cycle threshold. Markers of microvilli: ALP: alkaline phosphatase, SI: sucrase isomaltase. Markers of tight junctions: CLDN: claudin, OCLN: occludin, TJP1: tight junction protein 1 (ZO-1), MLCK: myosin light chain kinase. Statistical significance was tested by randomization tests using REST2009.

## References

1. Lesuffleur T, Barbat A, Dussaulx E, Zweibaum A: **Growth adaptation to methotrexate of HT-29 human colon carcinoma cells is associated with their ability to differentiate into columnar absorptive and mucus-secreting cells.** *Cancer Res* 1990, **50:**6334-6343.
2. Walter E, Janich S, Roessler BJ, Hilfinger JM, Amidon GL: **HT29-MTX/Caco-2 cocultures as an in vitro model for the intestinal epithelium: in vitro-in vivo correlation with permeability data from rats and humans.** *J Pharm Sci* 1996, **85:**1070-1076.
