# Supplementary material for: Titanium dioxide nanoparticle impact and translocation through ex vivo, in vivo and in vitro gut epithelia
Source: Part Fibre Toxicol. 2014 Mar 25;11:13. doi: 10.1186/1743-8977-11-13 (PMC3987106; doi:10.1186/1743-8977-11-13)
Supplement: Additional file 4 — NP interference with MTT assay. Interference is probed by addition of TiO2-NP to dissolved formazan crystals, and absorbance measurement. [file 1743-8977-11-13-S4.pdf]

Assessment of NP interference with MTT assay

To assess NP interference with MTT assay, control cells were incubated with a 0.5 mg/mL MTT solution. After 2 h at 37°C, the medium was removed and formazan crystals were dissolved in DMSO. To these solutions were then added 10-1000 µg/mL of TiO_2-NPs. After 5 min at 37 °C, the absorbance was read on either non-centrifuged or centrifuged plates. The results show that the interference of TiO_2-NPs with MTT is lowered (Figure 1). On wells exposed to 50 µg/mL NPs and centrifuged, the residual interference would lead to interpretation of a 2.25 ± 1.72% cell death, which is not statistically significant. Conversely on non-centrifuged wells, the misinterpretation would lead to a significant 5.47 ± 1.38% false-positive cell death. Metabolic activity was determined as a percentage of the negative controls (n=5).

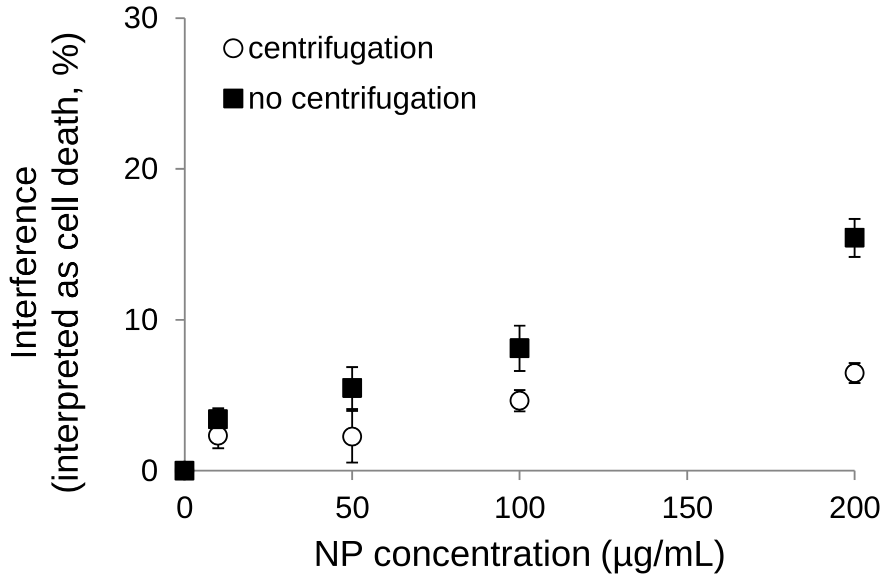

Figure 1. NP interference with MTT assay. Interference was probed by TiO_2-NP addition to dissolved formazan crystals.
