# Supplementary material for: Titanium dioxide nanoparticle impact and translocation through ex vivo, in vivo and in vitro gut epithelia
Source: Part Fibre Toxicol. 2014 Mar 25;11:13. doi: 10.1186/1743-8977-11-13 (PMC3987106; doi:10.1186/1743-8977-11-13)
Supplement: Additional file 5 — PIXE images of Ti accumulation in HT29-MTX monoculture cross-sections. PIXE images of cell cross-sections, showing that TiO2-NPs are really accumulated in the cells, and not adsorbed on the cell membrane. [file 1743-8977-11-13-S5.pdf]

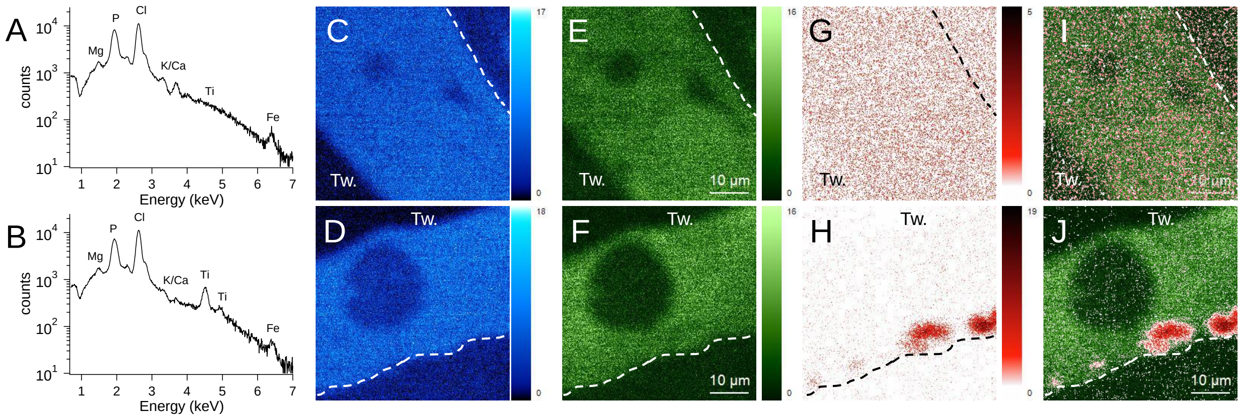

PIXE images of Ti accumulation in HT29-MTX monoculture cross-sections. PIXE spectra of the whole area (A-B) clearly showing a Ti-peak in TiO_2-NP exposed cells. Distribution of P (C-D), K/Ca (E-F), Ti (G-H), showing the cell delineation (P and K/Ca) and regions where TiO_2-NP are accumulated (Ti). Superimposition of K/Ca and Ti distribution images (I-J), showing that the Ti-rich regions are inside the cells, close to the apical pole (opposite to the transwell), and not adhering to the cell surface. PIXE analyses were performed on HT29-MTX cells not exposed to NPs (A-I), or exposed to 50 µg/mL of TiO_2-NPs for 24 h (B-J).
