# Supplementary material for: Titanium dioxide nanoparticle impact and translocation through ex vivo, in vivo and in vitro gut epithelia
Source: Part Fibre Toxicol. 2014 Mar 25;11:13. doi: 10.1186/1743-8977-11-13 (PMC3987106; doi:10.1186/1743-8977-11-13)
Supplement: Additional file 6 — Energy dispersive imaging and electron diffraction analyses of a TiO2-NP agglomerate inside a M-cell. This additional file shows TEM images and their EDS analysis, together with the electron diffractogram recorded on the electron-dense agglomerate of NPs located inside the M-cell and its analysis. [file 1743-8977-11-13-S6.pdf]

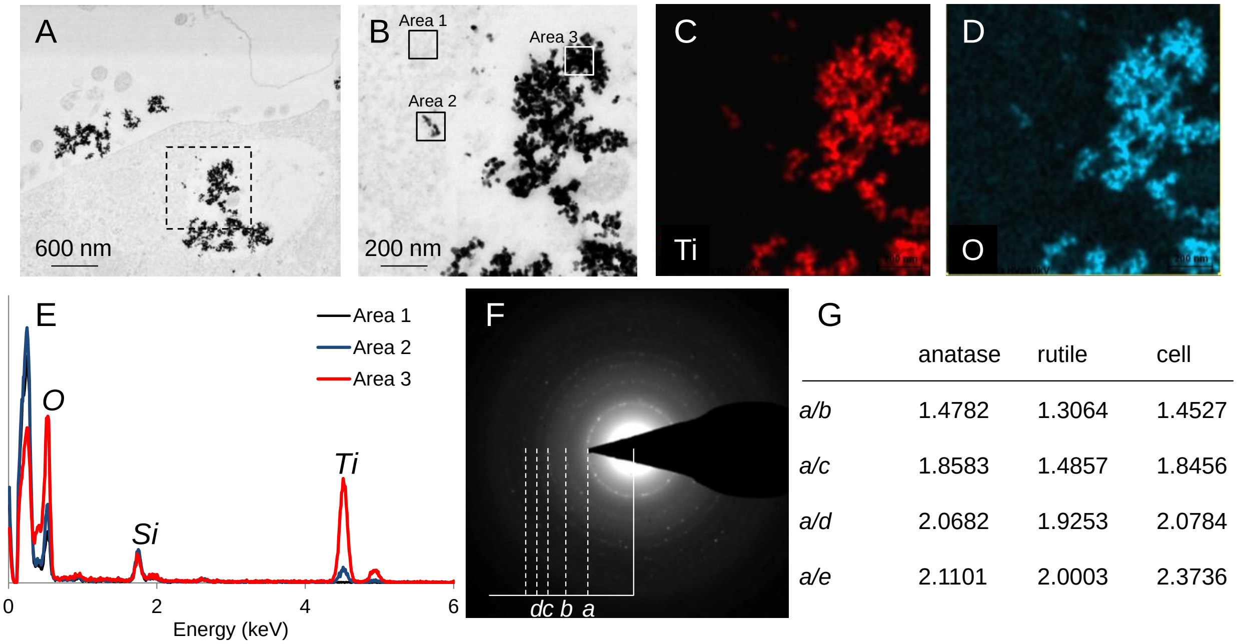

	anatase	rutile	cell
a/b	1.4782	1.3064	1.4527
a/c	1.8583	1.4857	1.8456
a/d	2.0682	1.9253	2.0784
a/e	2.1101	2.0003	2.3736

Transmission electron microscopic (TEM) observation, energy dispersive spectroscopy (EDS) and electron diffraction analyses of M-cells exposed to TiO_2-NPs. TEM image of a M-cell where TiO_2-NPs have been internalized and are stored in a cytoplasmic compartment (A), higher magnification image of the area defined in A (B). EDS imaging of Ti (C, red) and O (D, blue) distribution in B. EDS spectra recorded on the area 1, 2 and 3 as defined in B. Electron diffraction analysis of the area 3 defined in B (F) and analysis of this diffractogram by calculation of a/b, a/c, a/d and a/e inverse distance ratios (1/nm) and comparison with the diffraction lines of anatase and rutile references (G).
