# Supplementary material for: Titanium dioxide nanoparticle impact and translocation through ex vivo, in vivo and in vitro gut epithelia
Source: Part Fibre Toxicol. 2014 Mar 25;11:13. doi: 10.1186/1743-8977-11-13 (PMC3987106; doi:10.1186/1743-8977-11-13)
Supplement: Additional file 7 — M-cells crossing the transwell insert membrane. Three TEM images show M-calls crossing the transwell membrane, along with the nanoparticles they contain. [file 1743-8977-11-13-S7.pdf]

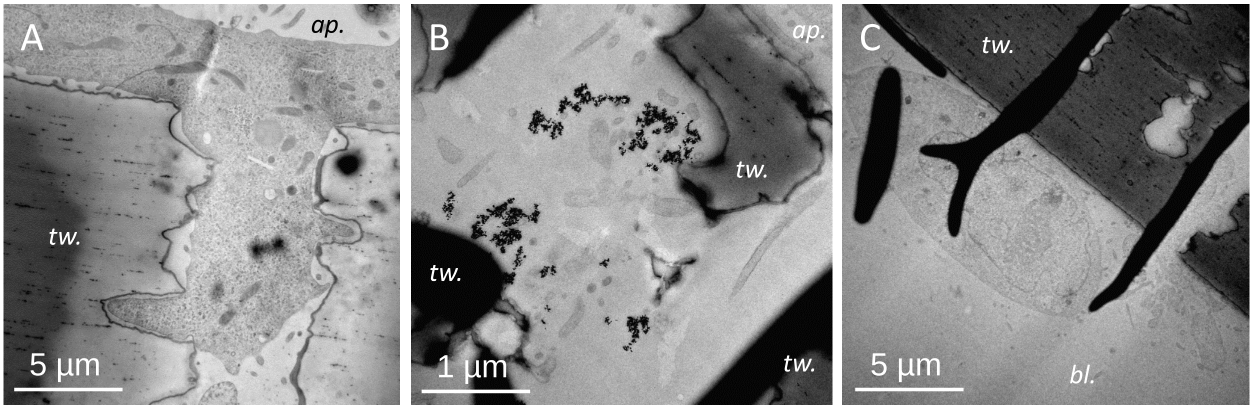

M-cells crossing the transwell insert membrane. When grown for 21 days on transwell inserts, Caco-2 cells co-cultured with RajiB lymphocytes were shown to disintegrate the transwell membrane and pass from the apical compartment to the basolateral compartment. This was observed both for unexposed cells (A, C) and for cells exposed to 50 µg/ml of TiO_2-NP for 48 h (B).
